# c-Mpl-del, a c-Mpl alternative splicing isoform, promotes AMKL progression and chemoresistance

**DOI:** 10.1038/s41419-022-05315-5

**Published:** 2022-10-13

**Authors:** Fei Li, Yuanyan Xiong, Mo Yang, Peiling Chen, Jingkai Zhang, Qiong Wang, Miao Xu, Yiming Wang, Zuyong He, Xin Zhao, Junyu Huang, Xiaoqiong Gu, Li Zhang, Rui Sun, Xunsha Sun, Jingyao Li, Jinxin Ou, Ting Xu, Xueying Huang, Yange Cao, Xiaohong Ruby Xu, Danielle Karakas, June Li, Heyu Ni, Qing Zhang

**Affiliations:** 1 State Key Laboratory of Biocontrol, School of Life Sciences, Sun Yat-sen University, Guangzhou, China; 2 The Seventh Affiliated Hospital, Sun Yat-sen University, Shenzhen, China; 3 Institute of Sun Yat-sen University in Shenzhen, Shenzhen, China; 4 Department of Laboratory Medicine and Pathobiology, University of Toronto, Toronto, Canada; 5 Department of Laboratory Medicine, Keenan Research Centre for Biomedical Science of St. Michael’s Hospital, and Toronto Platelet Immunobiology Group, University of Toronto, Toronto, Canada; 6 Canadian Blood Services Centre for Innovation, Toronto, Canada; 7 Department of Blood Transfusion, Clinical Biological Resource Bank and Clinical Lab, Guangzhou Institute of Pediatrics, Guangzhou Women and Children’s Medical Center, Guangzhou Medical University, Guangzhou, China; 8 State Key Laboratory of Oncology in South China, Sun Yat-sen University Cancer Center, Guangzhou, China; 9 National Key Clinical Department and Key Discipline of Neurology, The First Affiliated Hospital, Sun Yat-sen University, Guangzhou, China; 10 Department of Physiology, University of Toronto, Toronto, Canada

**Keywords:** Acute myeloid leukaemia, Acute myeloid leukaemia

## Abstract

Acute megakaryocytic leukemia (AMKL) is a clinically heterogeneous subtype of acute myeloid leukemia characterized by unrestricted megakaryoblast proliferation and poor prognosis. Thrombopoietin receptor c-Mpl is a primary regulator of megakaryopoeisis and a potent mitogenic receptor. Aberrant c-Mpl signaling has been implicated in a myriad of myeloid proliferative disorders, some of which can lead to AMKL, however, the role of c-Mpl in AMKL progression remains largely unexplored. Here, we identified increased expression of a c-Mpl alternative splicing isoform, c-Mpl-del, in AMKL patients. We found that c-Mpl-del expression was associated with enhanced AMKL cell proliferation and chemoresistance, and decreased survival in xenografted mice, while c-Mpl-del knockdown attenuated proliferation and restored apoptosis. Interestingly, we observed that c-Mpl-del exhibits preferential utilization of phosphorylated c-Mpl-del C-terminus Y607 and biased activation of PI3K/AKT pathway, which culminated in upregulation of GATA1 and downregulation of DDIT3-related apoptotic responses conducive to AMKL chemoresistance and proliferation. Thus, this study elucidates the critical roles of c-Mpl alternative splicing in AMKL progression and drug resistance, which may have important diagnostic and therapeutic implications for leukemia accelerated by c-Mpl-del overexpression.

## Introduction

Acute megakaryocytic leukemia (AMKL) is a heterogenous subtype of acute myeloid leukemia (AML) characterized by unrestricted proliferation of immature megakaryocytes (megakaryoblasts), and extensive myelofibrosis [1, 2]. Clinically, the disease is bimodally distributed with peaks in children between the ages 1 and 3 and adults ages 50 and 60 [3, 4]. AMKL in children is often strongly correlated with a genetic basis, with a better prognosis, particularly, in Down syndrome patients [5, 6]. In adults, the cytogenetic profile is diverse and the genetic link diffuse, as such the disease is less well understood [7, 8]. Importantly the prognosis is extremely poor and is considered an independent adverse prognostic factor for overall survival in AML [8, 9]. Leukemogenesis in AMKL can be de novo or secondary to myeloproliferative disorders, with a large proportion of patients possessing a normal karyotype, suggesting potential aberrancy at the molecular or protein level [10–12]. Therefore, mechanisms beyond cytogenetic abnormalities may constitute a significant basis for AMKL progression and thus warrants further research.

c-Mpl, a protooncogenic receptor, is constitutively expressed on megakaryocytes, platelets, and hematopoietic stem cells (HSCs) [13]. During normal hematopoiesis, via its ligand thrombopoietin (TPO), c-Mpl is a master regulator of megakaryopoiesis and platelet production as well as maintenance of the HSC niche [14, 15]. Pathologically, overactivation through activating mutations has been attributed to the development of myeloproliferative disorders, leading to secondary AMKL [16, 17]. In addition, increased expression of c-Mpl in CD34+ AML cells has been linked with poor prognosis, and improved maintenance of AML stem cell niche [18, 19]. Despite increasing evidence for the permissive role of c-Mpl in AML leukemogenesis, the functional role of c-Mpl in AMKL progression has not been adequately explored.

Alternative splicing of hematopoietic factor receptors plays an essential role in maintaining normal and pathologic hematopoiesis. These findings have been well described for the truncated erythropoietin receptor, where its expression markedly decreased in the blood cells of patients with polycythemia vera, and the co-expression of truncated and full-length forms of erythropoietin receptors develop the ability to transduce mitogenic signals [20, 21]. One regulatory mechanism of c-Mpl function is through alternatively spliced isoforms [22, 23]. In humans, four isoforms of c-Mpl have been identified including c-Mpl-p, c-Mpl-k, c-Mpl-tr, and c-Mpl-del, of which c-Mpl-p encodes the full-length 635 amino acid functional receptor [24, 25]. c-Mpl-k possesses an alternate intracellular domain and c-Mpl-tr is a prematurely truncated isoform, with potentially both acting as dominant-negative isoforms of the receptor [23, 26]. Previous studies also have shown that the c-Mpl-del isoform which encompasses a 24 amino acid deletion between exons 8 and 9 in the extracellular region was detected in CD34+ cells, megakaryocytes, and platelets isolated from healthy donors and essential thrombocythemia (ET) patients as well as megakaryocytic leukemia cells [27–29]. The presence of c-Mpl-del might reflect the stable adaptive regulation for megakaryocyte development and abnormal expansion of hematopoietic cells by having different roles in physiological and pathological functions. However, the relative expression levels of c-Mpl-del in megakaryoblastic leukemia cells are unknown partly due to the rarity of AMKL cases and the lack of quantitative and specific detection to distinguish other c-Mpl isoforms. This highlights the need for a renewed and precise approach to the prognosis and treatment of AMKL.

In this study, we observed a significant increase of c-Mpl-del in AMKL patients and identified a critical role for c-Mpl-del in AMKL progression, survival, and resistance to chemotherapy. These findings not only improve our understanding of AMKL progression but also uncover potential novel therapeutic targets and biomarkers in AMKL.

## Results

### c-Mpl-del, but not wild-type c-Mpl-p, is highly expressed in AMKL cells

**Fig. 1:**
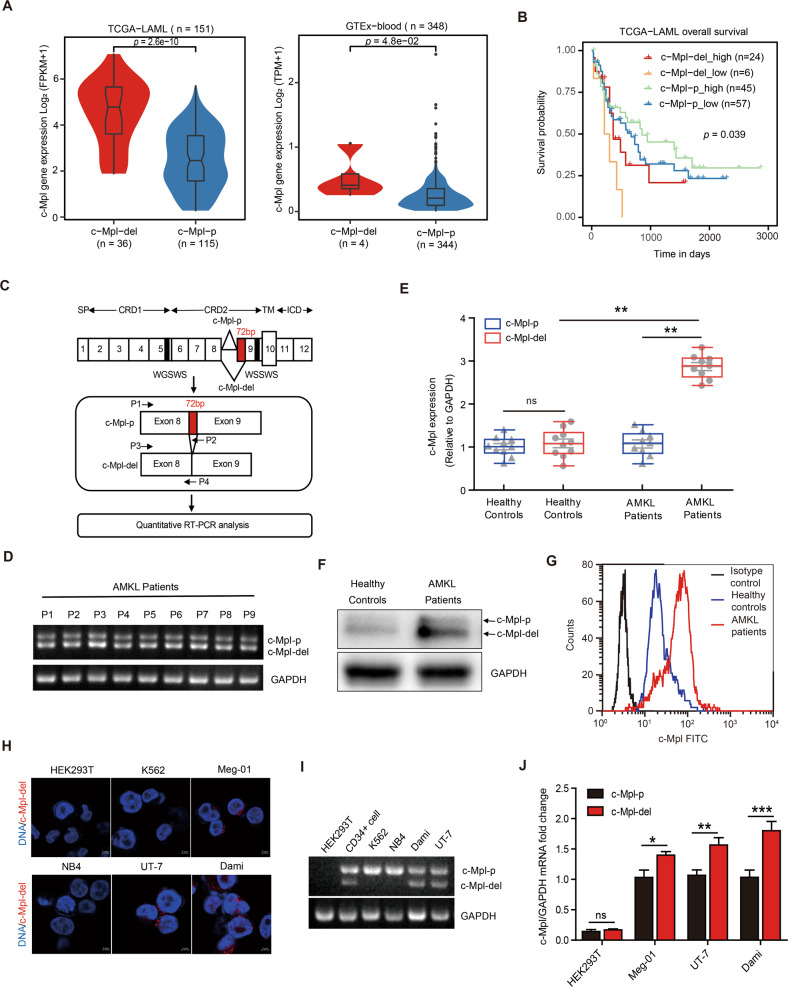
Increased expression of c-Mpl-del in AMKL cells and bone marrow cells of AMKL patients. **A** c-Mpl expression in GTEx-blood (*n* = 348) and TCGA-LAML (*n* = 151) samples with c-Mpl-del expression (c-Mpl-del group) or without c-Mpl-del expression (c-Mpl-p group). **B** Survival analyses of c-Mpl-del expressed AML patients and only c-Mpl-p expressed AML patients in TCGA data set (log-rank test *P* value = 0.039). **C** qRT-PCR primer design for deferential detection of c-Mpl-p and c-Mpl-del. The 72bp-deleted region of c-Mpl-del was indicated as a red box in the extracellular domain of this schematic diagram of exon maps of c-Mpl mRNA. The forward primers of c-Mpl-p and c-Mpl-del are the same (P1 and P3). The reverse primer of c-Mpl-p is specifically located upstream of exon 9 and the 3’ end of the 72 bp region (P2). The reverse primer of c-Mpl-del span exon 8 and exon 9 without 72 bp (P4). SP signal peptide, CRD cytokine receptor domain, TM trans-membrane, ICD intracytoplasmic domain. **D** Reverse transcription PCR (RT-PCR) analysis of c-Mpl-p and c-Mpl-del expression in bone marrow cells from AMKL patients (*n* = 9). **E** quantitative PCR analysis of c-Mpl-p and c-Mpl-del in bone marrow cells from AMKL patients (*n* = 9) and healthy donors (*n* = 10) relative to GAPDH. **F** Representative immunoblot of increased c-Mpl-del protein in bone marrow cells of AMKL patients compared to healthy controls. **G** Representative flow cytometry histogram of increased surface expression of total c-Mpl in AMKL single cell bone marrow aspirates compared to healthy control. **H** The subcellular distribution of c-Mpl-del transcripts was visualized by RNA FISH in HEK293T, K562, NB4, Meg-01, UT-7, and Dami (Scale bar 5 μm). **I** Reverse transcription PCR analysis of c-Mpl-p and c-Mpl-del expression in AMKL (Dami, UT-7) and non-megakaryocytic AML cell lines (K562, NB4), negative control HEK293T and healthy donor cord blood CD34+ cells. **J** quantitative PCR analysis of c-Mpl-p and c-Mpl-del in HEK293T, Meg-01, UT-7, and Dami cells relative to GAPDH. Data represent mean ± SD (*p < 0.05; **p < 0.01; ***p < 0.001).

c-Mpl-del expression originates from the alternative splicing of 72 bp between exons 8 and 9 in the extracellular region of c-Mpl [29]. To discover the expression of c-Mpl-del isoform and whether c-Mpl-del expression is correlated with the clinical-pathologic characteristics of AML patients, we firstly analyzed c-Mpl-del and c-Mpl-p expression in TCGA-LAML and GTEx-blood datasets and distinguished the c-Mpl-del (c-Mpl-del expressed samples) and c-Mpl-p (only c-Mpl-p expression, without c-Mpl-del expression) groups by extracting the splice junction reads in each BAM file (Fig. S1A). Notably, a total of 36 c-Mpl-del expressed AML patients (24%) exhibited significantly high expression of c-Mpl compared to the 115 AML patients without c-Mpl-del expression (Fig. 1A). However, only 4 of the 348 (one percent) blood samples from healthy people showed c-Mpl-del expression (Fig. 1A), suggesting that c-Mpl-del likely has an AML-biased expression. Meanwhile, AML patients with c-Mpl-del expression had shorter survival times than those without c-Mpl-del expression (Fig. 1B). Gene ontology (GO) annotation analysis of the differentially expressed genes (DEGs) between c-Mpl-del and c-Mpl-p samples for TCGA-LAML dataset also demonstrated that the c-Mpl-del expression involved in the regulation of multiple haematological physio-pathological processes (Fig. S1B). In our previous work, we had observed that the c-Mpl-del appears in human AMKL Dami cells, a subtype of AML, by targeting c-Mpl sequence, which could bind TPO and activate ERK1/2 signaling in c-Mpl-del-transfected NIH3T3 cells [27]. To identify the c-Mpl-del isoform within AMKL patients which were confirmed to contain blast hypercellularity (Fig. S1C), we performed qRT-PCR utilizing primers to specifically detect the expression of wild-type c-Mpl-p and splicing mutant c-Mpl-del (Fig. 1C). Amplification of cDNA yielded the full-length wild-type and truncated spliced variant isoform, corresponding to the c-Mpl-p and c-Mpl-del transcripts respectively, in bone marrow cells of AMKL patients (Fig. 1D). Importantly, c-Mpl-del but not c-Mpl-p exhibited significantly increased expression in AMKL patients compared to healthy controls at both the transcript and protein level (Fig. 1E, F). Notably, high expression of c-Mpl on the bone marrow cell surface of AMKL patients as determined by flow cytometry might imply that c-Mpl-del could be expressed on the cell membrane and mediate signal transduction (Fig. 1G). RNA fluorescence in situ hybridization (FISH) using splicing region-specific probes for c-Mpl-del and PCR amplification of cDNA corroborated that c-Mpl-del expressed in AMKL cell lines (Meg-01, UT-7, and Dami) (Fig. 1H, I). This was further verified with melting curve analysis and quantitative PCR that c-Mpl-del was elevated in AMKL cells Meg-01, UT-7, and Dami (Fig. 1J and Fig. S1D, E). Together, these data identify an increased expression of c-Mpl-del in AMKL patients and may potentially contribute to AMKL progression.

### Increased expression of c-Mpl-del enhances AMKL cells proliferation

**Fig. 2:**
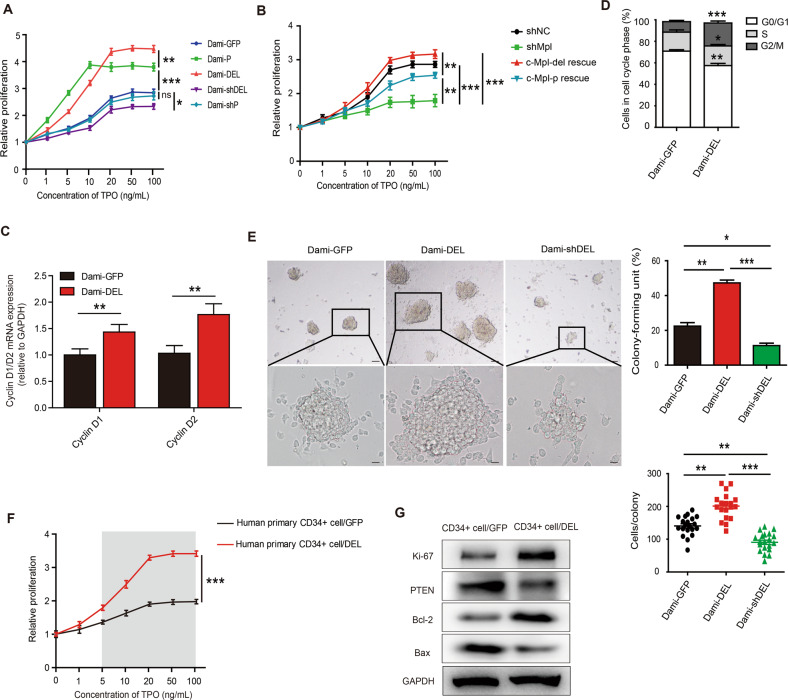
c-Mpl-del enhances AMKL and CD34+ proliferation in response to TPO. **A** Cell proliferation of c-Mpl-del (Dami-DEL), c-Mpl-p (Dami-P), control GFP (Dami-GFP), c-Mpl-p shRNA (Dami-shP), or c-Mpl-del shRNA (Dami-shDEL) transduced Dami cells 48 h following stimulation with various TPO concentrations as measured by CCK-8 cell proliferation assay. **B** Rescue assay showing proliferative activity of c-Mpl-del and c-Mpl-p rescue in Dami cells following knockdown of the endogenously expressed c-Mpl. c-Mpl-del or c-Mpl-p expressing lentivirus was used to transfect and rescue the expression of c-Mpl-del or c-Mpl-p. **C** Quantitative PCR analysis of relative cyclin D1 and D2 expression. **D** Cell cycle analysis and representative histograms of cells in the G0/G1, S, and G2/M phases as determined by 7-AAD staining of Dami-DEL and Dami-GFP 48 h following TPO (20 ng/mL) stimulation. **E** Representative micrographs of indicated transduced Dami colonies were assessed in methylcellulose cultures. Graphs represent the percentage of colony-forming units (# of clones/# of cells initially inoculated) (Scale bar 160 μm) and the number of cells/colony (calculated from 20 colonies in all experiments) (Scale bar 20 μm). Experiments were repeated three times. **F** Cell proliferation of freshly isolated human primary CD34+ cells transduced with control or c-Mpl-del-expressing lentivirus was measured with CCK-8 assay 48 h after the addition of the indicated TPO concentrations. **G** Representative immunoblot measuring expression levels of Ki-67, PTEN, Bcl-2, and Bax in freshly isolated human primary CD34+ cells transduced with control or c-Mpl-del expressing vector lentivirus 48 h following culture with TPO (20 ng/mL). Experiments were performed in triplicate. Data represent the mean ± SD (*n* = 3; *p < 0.05; **p < 0.01; ***p < 0.001).

To determine the functional role of c-Mpl-del expression in response to TPO simulation in AMKL, we overexpressed c-Mpl-p or c-Mpl-del in Dami and UT-7 cells that were termed Dami-P/UT-7-P, Dami-DEL/UT-7-DEL (Fig. S1E). We observed a dose-dependent proliferative response to TPO in both c-Mpl-p and c-Mpl-del transfected cell lines. However, interestingly, Dami-DEL exhibited higher proliferative potential compared to Dami-P, despite having similar levels of c-Mpl-del and c-Mpl-p overexpression (Fig. 2A, Fig. S1E). Conversely, c-Mpl-del shRNA targeted knockdown (Dami-shDEL), but not c-Mpl-p knockdown (Dami-shP), exhibited inhibited proliferation compared to control GFP-transfected cells (Dami-GFP) after c-Mpl-del or c-Mpl-p expression was successfully silenced by transfecting Dami cells with lentivirus-mediated shRNAs (Fig. 2A, Fig. S2A, B). Similar results were observed in an analogous AMKL cell line UT-7 (Fig. S2C). Meanwhile, although the rescue of c-Mpl-p expression following knockdown of the endogenously expressed c-Mpl restored the proliferative activity of Dami and UT-7 cells to some extent, the rescue of c-Mpl-del expression was more able to promote the proliferation of Dami and UT-7 cells under the same amount of rescue of c-Mpl-del and c-Mpl-p (Fig. 2B, Fig. S2D, E), indicating that c-Mpl-del has a stronger proliferative activity and TPO sensitivity in AMKL cells than c-Mpl-p. Further indicators of enhanced Dami-DEL proliferation included upregulation of cell cycle regulators cyclin D1 and cyclin D2 (Fig. 2C), complimentary with greater proportions of cells in active S and G2/M versus G0/G1 phases (Fig. 2D). In parallel, ex vivo colony formation in presence of TPO was markedly increased in Dami-DEL cells but was attenuated in Dami-shDEL cells (Fig. 2E). Interestingly, increased expression of the stem cell markers CD34 and CD38 was positively correlated with c-Mpl-del levels in Dami cells (Fig. S2F) suggesting that c-Mpl-del directly supports self-renewal of AMKL cells. Indeed, although human primary CD34+ cells endogenously express lower c-Mpl-del than c-Mpl-p (Fig. S1E), stable transfection of c-Mpl-del resulted in significantly higher proliferation in a TPO dose-dependent manner (Fig. 2F). This was consistent with proliferation marker Ki-67 upregulation and tumor-suppressor gene PTEN downregulation (Fig. 2G). Taken together these results demonstrate that c-Mpl-del confers an enhanced proliferative response of AMKL cells to TPO and putatively contributes to the maintenance of blast self-renewal and leukemogenesis.

### c-Mpl-del overexpression promotes chemotherapeutic drug resistance

**Fig. 3:**
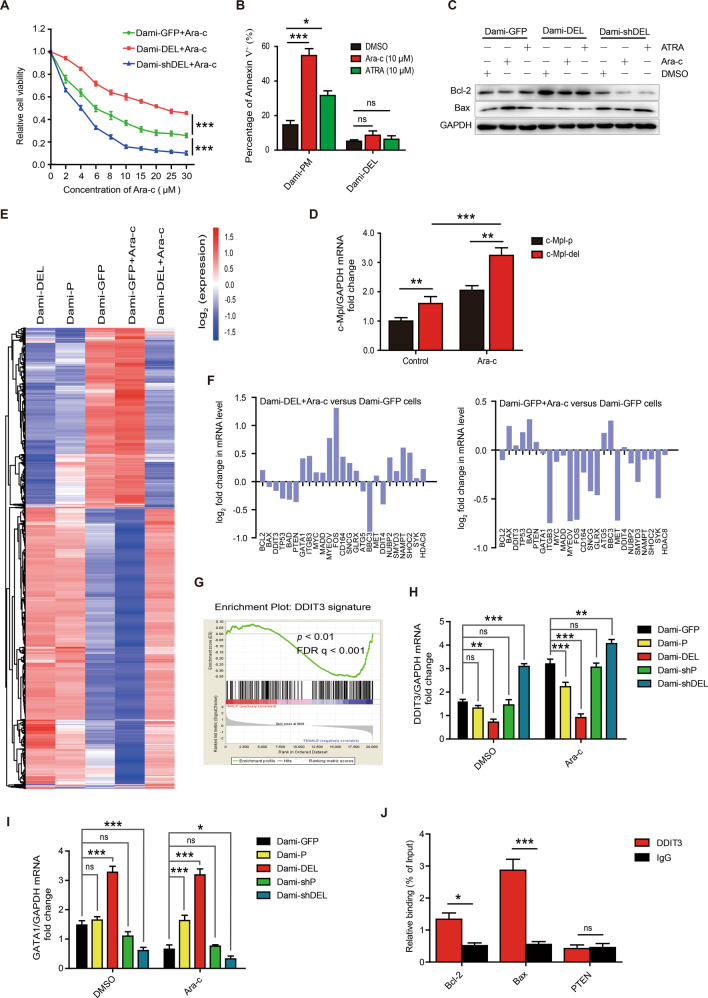
c-Mpl-del promotes chemotherapeutic drug resistance of AMKL cells. **A**–**C** Cell viabilities of differentially transfected Dami cells at 48 h post treatment of 20 ng/mL TPO with indicated concentrations of Ara-c treatment was measured using (**A**) CCK-8 viability assay and (**B**) Flow cytometric Annexin V binding. **C** Immunoblot analysis of Bcl-2 and Bax expression. **D** Quantitative PCR of c-Mpl-p and c-Mpl-del expression in Ara-c (10 μM) selected Dami cells versus non-treated Dami cells. **E** Heatmap displaying the hierarchical clustering of genes in Dami-DEL, Dami-P, and Dami-GFP cells in response to Ara-c treatment following exposure to 20 ng/mL of TPO. Gene expression values are displayed as progressively brighter shades of red (upregulated) or blue (downregulated). **F** mRNA cluster from RNA-seq demonstrating increased expression of survival genes in Dami-DEL following Ara-c treatment. **G** GSEA indicated significant proliferation-related gene enrichment classified by DDIT3 signature in Dami-DEL cells. **H** quantitative PCR analysis of DDIT3 expression in Dami-GFP, Dami-P, Dami-DEL, Dami-shP, and Dami-shDEL cells treated with or without Ara-c. **I** quantitative PCR analysis of GATA1 expression in Dami-GFP, Dami-P, Dami-DEL, Dami-shP, and Dami-shDEL cells treated with or without Ara-c. **J** The ability of DDIT3 binding to regulatory regions of the survival-associated genes was tested by ChIP-qPCR. ChIP-qPCR enrichment is shown as the percentage of input DNA. All experiments were repeated three times. Data represent the mean ± SD (*n* = 3; *p < 0.05; **p < 0.01; ***p < 0.001).

Similar to other AML subtypes, multidrug resistance and relapse remain the major obstacles in conventional AMKL treatment [3, 30]. Ara-c and ATRA are common cytotoxic chemotherapeutics in AML, however, a significant portion of patients do not respond well [18, 31]. We thus investigated the contribution of c-Mpl-del on AMKL chemotherapeutic drug resistance. We found increased apoptotic resistance and cell viability in c-Mpl-del overexpressing AMKL cells across various doses of Ara-c and ATRA (Fig. 3A, Fig. S3A). Conversely, c-Mpl-del knockdown reduced the viabilities of AMKL cells compared to control vector-transfected cells (Fig. 3A). In addition, in puromycin (PM) selected drug-resistant prone Dami cell lines, Ara-c and ATRA maintained increased apoptosis induction by two to three-fold in control Dami-PM but was completely abrogated in Dami-DEL when c-Mpl-del was overexpressed (Fig. 3B). Concomitantly distinct inverse levels of upregulated pro-survival Bcl-2 and downregulated pro-apoptotic Bax was not altered by Ara-c or ATRA treatment in Dami-DEL cells. In contrast, Ara-c treatment decreased Bcl-2 and increased Bax in control AMKL cells, with the greatest response seen in c-Mpl-del shRNA knockdown (Dami-shDEL) (Fig. 3C, Fig. S3B). Moreover, in Ara-c enriched resistant AMKL cells, c-Mpl-del expression significantly increased (Fig. 3D), which suggests a feed-forward response in c-Mpl-del mediated AMKL drug resistance.

We then performed high throughput RNA-seq from Ara-c- treated Dami-DEL and Dami-GFP cells to identify in-depth the differential genes or critical pathways that are activated. The majority of anti-apoptotic genes (such as Bcl-2 and Myc) were upregulated in Dami-DEL cells. In contrast, the expression of pro-apoptotic gene (such as Bax and Bad) were significantly downregulated by the overexpression of c-Mpl-del. We also saw a shift towards pro-apoptotic gene sets in Ara-c-treated control Dami-GFP cells, including the upregulation of Bax and Bad, which was inversed in Ara-c-treated Dami-DEL cells (Fig. 3E, F and Fig. S3C). Notably, gene set enrichment analysis (GSEA) revealed a highly significant enrichment of negatively correlated DNA damage-inducible transcript 3 protein (DDIT3) genes sets and positively correlated GATA1 in Dami-DEL cells compared to Dami-GFP cells (Fig. 3G, Fig. S3D), both of which have been reported to be involved in the regulation of hematopoiesis and pathogenesis of leukemia, respectively [32, 33]. The qRT-PCR analysis further confirmed the downregulation of DDIT3 and the upregulation of GATA1 in Dami-DEL and UT-7-DEL cells, which was reversed by the transfection of c-Mpl-del-specific shRNAs but not c-Mpl-p-specific shRNAs. Meanwhile, under Ara-c treatment, DDIT3 was upregulated and GATA1 was downregulated in Dami-GFP and UT-7-GFP cells, but this regulation was counteracted by the overexpression of c-Mpl-del (Fig. 3H, I, and Fig. S3E, F), indicating that c-Mpl-del could antagonize Ara-c and downregulate DDIT3 and upregulate GATA1 in AMKL cells. Using CentriMo for motif enrichment analysis of GATA1 from JASPAR database, we also found that GATA1 was significantly positively correlated with c-Mpl-del expression in AML patients (Fig. S3G). In addition, using Kyoto Encyclopedia of Genes and Genomes (KEGG) pathway analysis, several major signal transduction pathways, including downstream TPO signaling genes, and c-Mpl transcriptional targets, and PI3K/AKT pathways, were modulated by Ara-c in control AMKL cells but blunted in cells with c-Mpl-del overexpression (Table S1, S2). Given that our datamining efforts identified DDIT3 and GATA1 as putative transcription factors in response to Ara-c, we next tested the ability of DDIT3 to directly bind to the promoter region of known survival-associated genes including the Bax, Bcl-2, and PTEN by chromatin immunoprecipitation-quantitative PCR (ChIP-qPCR). We found that the DDIT3 protein was able to bind the upstream promoter of Bax and Bcl-2 except for PTEN (Fig. 3J). This supports the notion that the apoptosis-related gene expression was controlled by the transcription factor DDIT3. Thus, these findings suggest TPO-stimulation drives a transcriptional program by which the downregulation of DDIT3 and upregulation of GATA1, skew towards survival and proliferation of c-Mpl-del-expressing AMKL cells.

### TPO/c-Mpl-del-activated PI3K/AKT pathway promotes anti-apoptotic responses

**Fig. 4:**
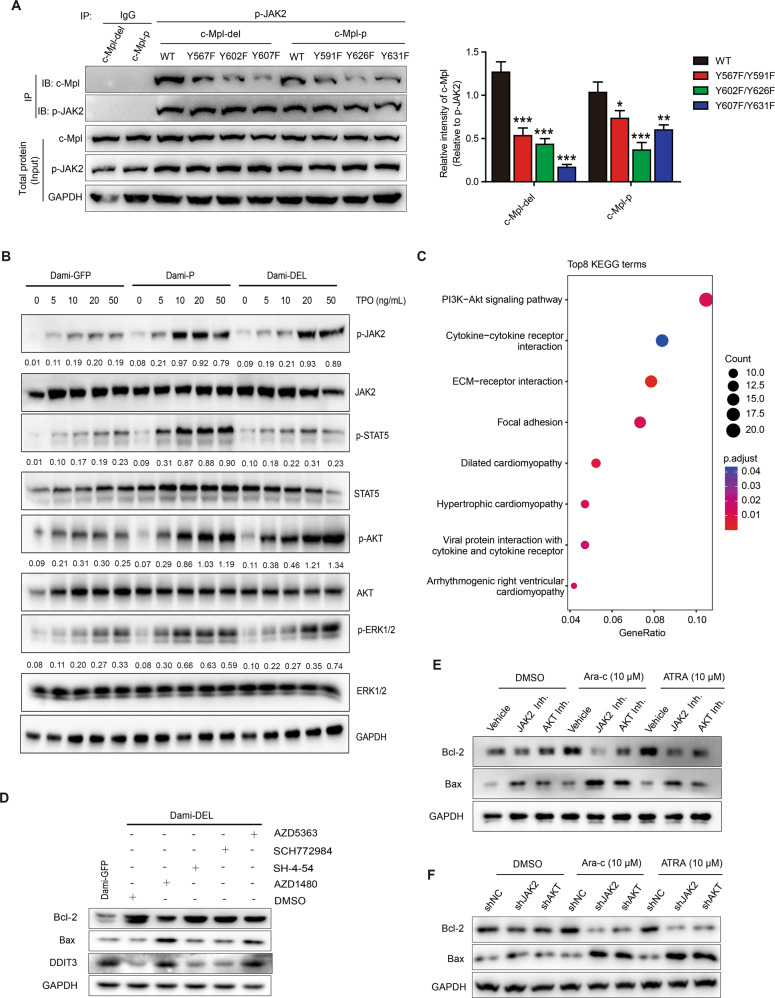
TPO/c-Mpl-del-activated PI3K/AKT pathway modulates anti-apoptotic responses. **A** Immunoblot and densitometry of HEK293T expressing c-Mpl C-terminus tyrosine mutants (Y567F, Y602F, Y607F for c-Mpl-del; Y591F, Y626F, Y631F for c-Mpl-p) co-immunoprecipitated with phospho-JAK2 following 20 ng/mL of TPO stimulation. **B** Immunoblot showing levels of phosphorylated downstream signaling proteins following Dami-P or Dami-DEL stimulation with indicated TPO concentrations. GAPDH was used as the loading control. Phospho-JAK2/GAPDH, phospho-AKT/GAPDH, phospho-STAT5/GAPDH, and phospho-ERK1/2/GAPDH densitometric ratios were recorded. **C** KEGG pathway enrichment analysis in c-Mpl-del expressed AML patients from TCGA data set. **D** Immunoblot showing levels of Bcl-2, Bax, and DDIT3 from Dami-GFP or Dami-DEL cells stimulated with TPO and treated with or without small molecule inhibitors of JAK2 (AZD1480), STATs (SH-4-54), ERK1/2 (SCH772984) and AKT (AZD5363). **E** Immunoblot showing the rescue of decreased Bcl-2 and increased Bax following Ara-c and ATRA treatment in presence of JAK2 (AZD1480) and AKT (AZD5363) inhibitors in TPO-stimulated Dami-DEL cells. **F** Immunoblot showing the rescue of decreased Bcl-2 and increased Bax following JAK2 and AKT knockdown in presence of Ara-c and ATRA in TPO-stimulated Dami-DEL cells. Data represent the mean ± SD (*n* = 3; *p < 0.05; **p < 0.01; ***p < 0.001).

To further investigate the mechanism by which c-Mpl-del signaling regulates and promotes AMKL cell survival and proliferation, we systematically generated and expressed tyrosine substitution mutants (Y591F/Y567F, Y626F/Y602F, and Y631F/Y607F) within the c-Mpl-p and c-Mpl-del cytoplasmic domain in HEK293T cells. We found lower levels of recovered tyrosine-mutants after immunoprecipitation of phosphorylated JAK2 indicating that all Y591/Y567, Y626/Y602 and Y631/Y607 residues within both c-Mpl-p and c-Mpl-del participated in TPO mediated signaling (Fig. 4A). However, we found in c-Mpl-del Y607F mutation TPO-stimulated tyrosine phosphorylation was particularly attenuated, indicating that Y607 is a key tyrosine residue in JAK2-mediated TPO/c-Mpl-del signaling (Fig. 4A). Phosphorylation analysis of downstream signaling molecules in overexpressing c-Mpl-p or c-Mpl-del Dami cells treated with TPO revealed subtle differences and signaling biases, with increased p-AKT in Dami-DEL and increased p-STAT5 and p-JAK2 in Dami-P (Fig. 4B), which was further confirmed by the KEGG analysis of c-Mpl-del-expressed samples for TCGA-LAML dataset (Fig. 4C). Thus, while c-Mpl-p-mediated TPO signaling activate JAK2/STAT5 pathway, c-Mpl-del-mediated TPO signaling may incline towards the PI3K/AKT pathway potentially through preferential phosphorylation of Y607 residue.

We next inhibited TPO/c-Mpl-del signaling pathways in Dami-DEL cells with small-molecule inhibitors of JAK2 (AZD1480), STATs (SH-4-54), ERK1/2 (SCH772984) and AKT (AZD5363) to assess its effect on AMKL viability. As expected, AZD1480 and AZD5363, but not SH-4-54 and SCH772984, decreased Bcl-2 expression and upregulated Bax and DDIT3 expression in Dami-DEL cells (Fig. 4D), indicating that the TPO/c-Mpl-del signaling primarily regulates apoptosis-related proteins and transcription factors via the JAK2/PI3K/AKT pathway. Notably, although Dami-DEL cells were resistant to Ara-c and ATRA alone, in the presence of AZD1480 or AZD5363, the pro-apoptotic effect was rescued with decreased Bcl-2 and increased Bax (Fig. 4E). Lentiviral shRNAs transfection also confirmed that c-Mpl-del-mediated Bcl-2 upregulation and Bax downregulation in the presence of Ara-c and ATRA were abolished by JAK2 or AKT knockdown in Dami-DEL cells (Fig. 4F and Fig. S4A). Meanwhile, both Bcl-2 knockdown and Bax overexpression counteracted the antiapoptotic effect of Dami-DEL cells under the treatment of Ara-c and ATRA (Fig. S4B), indicating that c-Mpl-del-mediated anti-apoptotic responses in AMKL cells through JAK2/PI3K/AKT pathway is fully dependent by increased Bcl-2 and decreased Bax.

### c-Mpl-del leads to increased malignant AMKL proliferation in xenograft mice

**Fig. 5:**
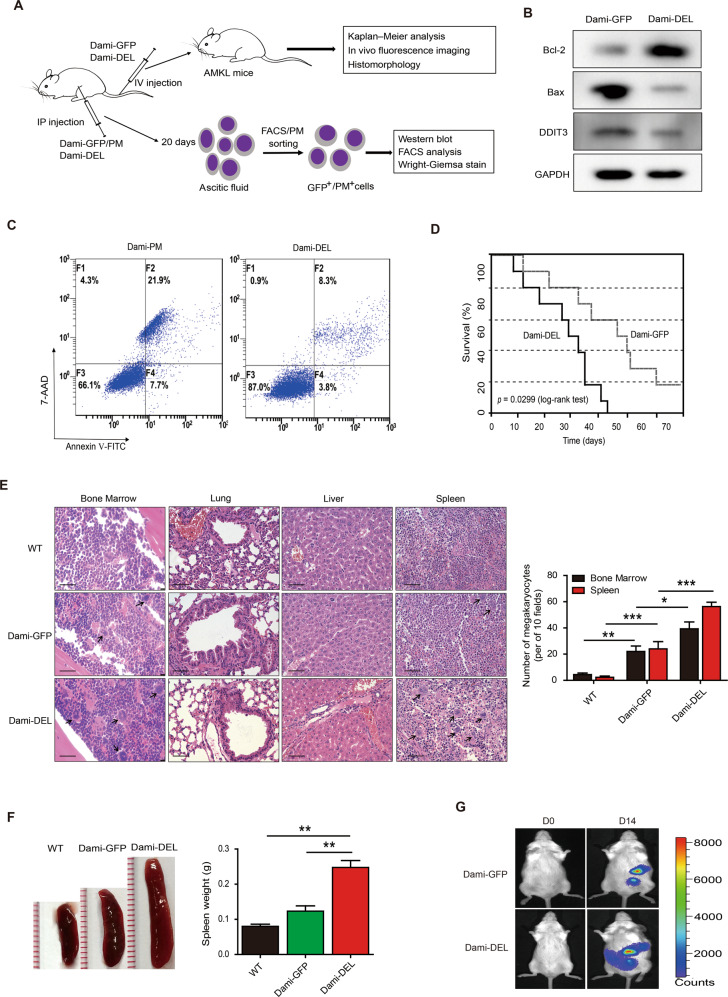
Overexpression of c-Mpl-del leads to increased malignant AMKL proliferation in xenograft mice. **A** Experimental design of mouse in vivo AMKL tumorigenesis (intraperitoneal injection (IP), ascites formation) and metastatic model (intravenous injection (IV)). **B** Immunoblot showing levels of Bcl-2, Bax, and DDIT3 in Dami-GFP and Dami-DEL ascitic cells harvested 20 days post engraftment. **C** Representative flow cytometry dot plot showing pro-apoptotic Annexin V and 7-AAD double staining of Dami-PM and Dami-DEL ascitic cells sorted by puromycin. **D**–**G** Two weeks following IV injection of Dami-GFP and Dami-DEL cells (**D**) mortality was assessed via Kaplan–Meier survival curves. *P* values were determined by the log-rank (Mantel–Cox) test, *n* = 10/group. **E** Histological analysis of the bone marrow, lung, liver, and spleen tissues. Arrows indicate diffuse infiltration of AMKL cells. Scale bar represents 40 µm. Graph shows the absolute megakaryocyte numbers counted in histological analysis at 10 high-power fields of view (magnification ×400) for each mouse. **F** Representative photos of spleen size and spleen weights. **G** Whole-body in vivo imaging showing infiltration and tumorigenesis of Dami-GFP-Luciferase and Dami-DEL-Luciferase IV injected mice over period of 2 weeks. Data represent the mean ± SD (*n* = 3; *p < 0.05; **p < 0.01; ***p < 0.001).

Lastly, to establish the in vivo significance of c-Mpl-del expressing AMKL cells in tumorigenesis and metastasis, we established both an AMKL-ascites and intravenous metastasis mouse model by injection of stable GFP/PM- and luciferase-expressing Dami/Dami-DEL cells into the abdominal cavity or tail vein of NOD/SCID mice (Fig. 5A). In AMKL tumorigenesis model, cells harvested from Dami-DEL established ascites were larger with poly-lobulated nuclei and less apoptotic as evidenced by increased Bcl-2, decreased Bax and DDIT3 compared to control Dami-GFP/Dami-PM (Fig. 5B, C and Fig. S5A).

We further addressed the dynamic in vivo effects of c-Mpl-del on AMKL proliferation and infiltration in a metastatic model. Overall, mice injected with Dami-DEL cells had increased mortality compared with Dami-GFP injected mice (Fig. 5D). Moreover, measurement of fluorescence intensity in circulation over the course of one month following IV injection demonstrated Dami-DEL-GFP cells underwent increased proliferation (Fig. S5B, C). Histological analysis verified that c-Mpl-del-overexpressing AMKL cells had significantly infiltrated the bone marrow, in addition to causing a complete loss of the pulmonary and hepatic architecture, including vascular structures and germinal centers (Fig. 5E). Similar findings were also observed in the spleen, wherein AMKL cell infiltration was observed, resulting in damage to the spleen architecture with marked splenomegaly and increases in megakaryocyte numbers (Fig. 5E, F).

In vivo imaging of mice confirms increased splenic migration and tumor establishment at 14 days post IV injection of Dami-DEL (Fig. 5G). Altogether, these in vivo findings demonstrate that higher c-Mpl-del expression is significantly correlated with rapid tumor growth, increased AMKL metastasis, and shorter survival which underscore the importance of c-Mpl-del-regulation of atypical megakaryoblasts in leukemogenesis.

## Discussion

AMKL is clinically challenging subtype of AML with poor prognosis and a not well understood pathogenesis, particularly in adults [2, 3, 34]. Here we elucidated a role of c-Mpl in supporting in AMKL malignancy, where we identified a novel function of a c-Mpl isoform c-Mpl-del that was found to be highly expressed in AMKL cells and bone marrow cells from AMKL patients. Such that, c-Mpl-del confers enhanced AMKL proliferation, survival and chemotherapeutic resistance by mediating TPO signaling, leading to a more aggressive disease in vivo.

The c-Mpl-del isoform arises from a 72 bp deletion which corresponds to a lack of 24 amino acids within the second cytokine receptor motif near the transmembrane domain [29]. Previous studies show during normal hematopoiesis, c-Mpl-del expression is limited to hematopoietic cells of the megakaryocyte lineage with relative expression level to c-Mpl-p increased during late megakaryopoesis with high expression in platelets [28]. Here, we performed a comprehensive analysis of the expression levels of c-Mpl-del and c-Mpl-p, by analyzing 499 RNA sequencing samples from TCGA AML and GTEx normal blood. We found that the expression pattern of c-Mpl-del and c-Mpl-p were significantly different in cancer and normal, showing tumor-specific high expression, and c-Mpl-del showed tumor-specific expression (Fig. 1A). We further identified and experimentally validated that c-Mpl-del expression levels were significantly correlated with the prognostic value. Our finding of increased ratio of c-Mpl-del to c-Mpl-p in AMKL also reveals a previously unidentified aspect of c-Mpl-del in the regulation of proliferation, viability and chemotherapeutic resistance suggesting the abnormal expression of c-Mpl-del may contribute to neoplastic events. Notably, in the analysis of TCGA-LAML data, although AML patients with c-Mpl-del expression had shorter survival times than those without c-Mpl-del expression, the patients expressing low c-Mpl-del in AML appeared to have a lower survival than those with high (Fig. 1B). While the difference does not reach statistical significance, it implies that there may be some AML subtype with a shorter survival period in the analysis of total AML, leading to the emergence of lower c-Mpl-del-expressed patients in total AML have a lower survival. In the future, further refinement of the analysis of AML subtypes to reveal the impact of c-Mpl-del on single AML subtypes other than AMKL may have important significance for exploring the pathogenesis of c-Mpl-del-related diseases.

It is not clear why there is an increased utilization of alternative splice sites in AMKL, leading to increased ratio of c-Mpl-del to c-Mpl-p. Although previously, RNA binding motif protein 15 (RBM15), first identified in translocation t(1:22)(p13;q13) AMKL, was reported to be an important regulator in maintaining full length c-Mpl-p expression through epigenetic regulation and direct mRNA spliceosome recruitment [23, 35, 36]. RBM15 knockdown resulted in increased generation of c-Mpl-tr splice variant isoform in HSCs and c-Mpl-del in Meg-01 cells [23, 36]. Whether there is similar dysregulation of spliceosome function and the potential mechanisms of RBM15 involved in regulating the splicing of c-Mpl-del may be worthwhile to be addressed in the future. In addition, by comparing the c-Mpl-del group with c-Mpl-p group in TCGA-LAML dataset, we found that the high expression of c-Mpl was accompanied by a high level of RUNX1T1 in AML patients (Fig. S6). RUNX1T1 is a RUNX1 partner transcriptional co-repressor 1 and fusion with RUNX1 to jointly regulate the mutation and abnormal expression of a large number of genes in AML, including RBM15 and c-Mpl [19, 37, 38]. This implies that the aberrant expression of RUNX1T1 may be one of the causes of RBM15 mutation and c-Mpl deregulation in AML, which further lead to the high formation of c-Mpl-del.

We observed biased activation of the pro-survival PI3K/AKT pathway particularly at lower levels of TPO (5 ng/mL) (Fig. 4B). It has been previously noted that there is a biphasic pro-survival and proliferation response within the c-Mpl-TPO axis, with a lower threshold of c-Mpl signaling preferentially activating the pro-survival PI3K/AKT followed by a stronger signal induction of proliferative RAS/ERK pathway [39]. Functionally, this is consistent with our observation of a lower proliferative response at lower doses of TPO (Fig. 2A). However, at higher doses of TPO, the switch to enhanced proliferation led to sustained TPO signaling and stronger PI3K/AKT signaling magnitude. As previously reported, the activated PI3K/AKT pathway induced GATA1 upregulation and DDIT3 downregulation [40, 41], and the PI3K/AKT pathway activated by TPO/c-Mpl-del also resulted in the upregulation of proliferation-promoting transcription factor GATA1 and the downregulation of DDIT3, leading to the upregulation of proliferative genes as well as antiapoptotic proteins in AMKL. A more in-depth study on the difference and mechanisms of c-Mpl-p and c-Mpl-del in the preferential activation of signal pathways may be of great significance to analyze the difference between the proliferation and apoptosis in AMKL caused by specific isoforms.

With regards to the downstream of c-Mpl-del signaling pathways, we have determined that downregulation of DDIT3 is required for AMKL progression by c-Mpl-del engagement controls apoptosis both in vitro and in AMKL tumorigenesis NOD/SCID mode (Figs. 3 and 5). This finding is in keeping with the previous reports that DDIT3 helps induce apoptosis in chemosensitive AML, and is suppressed by the loss of RUNX1 methylation identified in familial leukemia [42, 43]. Interestingly, the upregulation of GATA1 and down-regulation of DDIT3 may have overlapping functions resulting in PI3K/AKT-related culminating survival signaling based on gene set enrichment analysis in response to Ara-c (Fig. 3G, Fig. S3D). The previous finding is that AKT collaborates with GATA1 to dysregulate megakaryopoiesis, locking megakaryocytes in an undifferentiation state and promoting AMKL [44]. Although GATA1 has also been associated with decreased sensitivity to Ara-c treatment [45, 46], further studies are warranted to determine whether c-Mpl-del upregulation of GATA1-associated genes could also increase resistance to a new class of AMKL therapeutics such as Aurora Kinase A that induces megakaryocyte polyploidization and differentiation [1]. Moreover, by investigating the downstream effector molecules differentially and mutually controlled by DDIT3 and GATA1, we may determine molecular events broadly important for AMKL malignancy and chemoresistance.

Lastly, when we recapitulated the enhanced proliferative and anti-apoptotic effects of c-Mpl-del on AMKL in an in vivo murine model of AMKL tumorigenesis and metastasis, we observed increased mortality and malignancy (Fig. 5). Although this model could not capture the leukemogenesis potential of c-Mpl-del expression, our in vitro data demonstrate overexpression of c-Mpl-del in human CD34+ stem cells increase proliferation and decreases apoptosis (Fig. 2F, Fig. S1E). As it has been previously demonstrated that increased c-Mpl expression of CD34+ cells increases leukemia potential [18, 47, 48], it remains to be determined in future clinical studies if the increased c-Mpl is predominantly of the c-Mel-del isoform.

In summary, this study is the first to identify an abnormal increase of c-Mpl-del expression in AMKL patients, which was further elucidated and found to confer increased malignancy and chemotherapeutic resistance to AMKL cells. Although it is clear that c-Mpl is a key contributor to AMKL progression, broad therapeutic targeting of c-Mpl may not be a desirable approach as it may disrupt the hemostatic HSC niche [49]. Thus, our identification of c-Mpl-del and associated downstream signaling pathways may be exploited as distinct therapeutic targets specific for megakaryocytic leukemia cells. Future studies will be required to assess the prognostic value of c-Mpl-del in AMKL patients and whether its expression level can be further correlated with refractoriness or relapse.

## Materials and methods

### Cell lines and AMKL samples

Human Meg-01, Dami, UT-7, and HEK293T cells were obtained from ATCC and were cultured in Iscove’s Modified Dulbecco Medium (HyClone, USA) and Dulbecco’s Modified Eagle’s Medium (Gibco, USA) supplemented with 10% fetal bovine serum (Gibco, USA), respectively. The identities of all four cell lines were validated by short tandem repeat profiling analysis or karyotype analysis. All clinical samples were obtained with informed consent from Sun Yat-sen University Cancer Center and approved by the Hospital’s Protection of Human Subjects Committee. The clinicopathological characteristics of the patients were summarized in Table S3. Detailed methods of human sample preparation are found in supplementary methods.

### RNA-seq dataset analysis

RNA-sequencing data of TCGA-LAML cohort were achieved through the Cancer Genome Atlas portal (https://portal.gdc.cancer.gov/). GTEx-blood RNA-seq raw reads and sample information were downloaded from the dbGaP database (phs000424.v6.p1).Clinical data downloaded by TCGAbiolinks [50] including the survival status, survival time, stages, histology subtype, gender, and race. To distinguish the c-Mpl-p and c-Mpl-del groups in LAML, the BAM file was extracted by samtools and the reads with MAPQ (mapping quality) ≥30 were reserved [51]. For each BAM file, the junction reads spanning exons 8 and 9 of c-Mpl gene were extracted. The number of c-Mpl-del specific isoform reads was obtained according to the CIGAR value (c-Mpl-del junction reads, c-Mpl-del contains 1980N, c-Mpl-p contains 1908N). The samples with the number of c-Mpl-del junction reads >0 were divided into c-Mpl-del group, and the other samples without this specific splicing isoform were divided into c-Mpl-p group. The data were analyzed with the R (version 4.1) and R Bioconductor packages. To identify DEGs in c-Mpl-del group against c-Mpl-p group for TCGA-LAML dataset, R package DESeq2 was applied to determine DEGs [52]. The significance criteria for determining DEGs was set as |log2FC|>1 and *p* < 0.05. The list of identified DEGs was summarized in Table S4. GO and KEGG annotation was performed by R package ClusterProfiler [53].

### Cell transfection and lentivirus-mediated gene knockdown

Lipofectamine 3000 reagent (Invitrogen, USA) was utilized for the transient transfection of HEK293T cells according to the manufacturer’s protocol. Stable cell lines were generated by transfecting Dami and UT-7 cells with control or c-Mpl-del/c-Mpl-p-expressing lentivirus generated with empty vectors or pCDH-CMV-Mpl-del/Mpl-p-EF1-copGFP/pCDH-CMV -Mpl-del/Mpl-p-EF1-puro lentiviral expression vectors with psPAX2 and pMD2.G in HEK293T cells as previously reported [54, 55]. The knockdown lentiviruses expressing negative control shRNA (shNC), shRNA targeting c-Mpl, shRNAs specifically targeting the alternative splicing region of c-Mpl-del (shMpl-del) and c-Mpl-p (shMpl-p), and shRNAs targeting JAK2 (shJAK2), AKT (shAKT), and Bcl-2 (shBcl-2) were constructed using the pLKO.1. The sense sequences of the oligonucleotide for all shRNAs were listed in Table S5. To confirm target knockdown, cells were collected for qRT-PCR or Western blot analysis. Detailed methods of in vitro assays are found in supplementary methods.

### Animal models

Five-week-old NOD/SCID mice were maintained in the Laboratory Animal Center of Sun Yat-sen University and procedures were performed according to the institutional ethical guidelines for animal experiments. The animal studies were authorized by the Institutional Animal Care and Use Committee of Sun Yat-sen University. The mice were randomized into different groups and used to generate AMKL xenograft mice by injecting 1 × 10^6^ stable GFP or PM-expressing Dami/Dami-DEL cells into the abdominal cavity of NOD/SCID mice, or injecting 1 × 10^6^ stable luciferase-expressing Dami/Dami-DEL cells into the tail vein as previously reported standard techniques [56]. Detailed methods of animal model assays are found in supplementary methods.

### Statistical analysis

All data were reported as the mean ± SD of three independent experiments. To compare two independent groups, two-tailed student’s *t* test or Wilcoxon signed-rank tests were performed per parametric and non-parametric independent metrics, respectively. *P* was set at 0.05 for rejecting null hypotheses. To compare more than two groups, all data were first analyzed to determine whether they adhered to a normal distribution and then subjected to one-way analysis of variance followed by Tukey’s post hoc test. Survival analysis for TCGA-LAML data was performed using R package survminer and a log-rank test was used to determine the statistical significance of differences. For mouse Kaplan–Meier survival curves, *P* values were produced using a log-rank (Mantel–Cox) test. *P < 0.05, **P < 0.01, and ***P < 0.001 were considered statistically significant.

## Supplementary information

aj-checklist

Supplementary materials

Supplementary Table S4

Supplementary Table S7

Original western blots

Author Contribution Statement

## Data Availability

All data generated or analyzed during this study are included either in this article or in the supplemental materials files. Additional raw data may be available from the corresponding author for reasonable reasons. The RNA-seq raw data have been deposited under the Gene Expression Omnibus (GEO) accession numbers GSE127762 and GSE145949.
