# Supplementary material for: c-Mpl-del, a c-Mpl alternative splicing isoform, promotes AMKL progression and chemoresistance
Source: Cell Death Dis. 2022 Oct 13;13(10):869. doi: 10.1038/s41419-022-05315-5 (PMC9561678; doi:10.1038/s41419-022-05315-5)
Supplement: Supplementary file 2 — Supplementary materials [file 41419_2022_5315_MOESM2_ESM.pdf]

Supplementary materials

c-Mpl-del, a c-Mpl alternative splicing isoform, promotes AMKL progression and chemoresistance

Fei Li[*1], Yuanyan Xiong[*1], Mo Yang[*2], Peiling Chen[1], Jingkai Zhang[1], Qiong Wang[1,3], Miao Xu[4,5], Yiming Wang[4,5,6], Zuyong He[1], Xin Zhao[1], Junyu Huang[1], Xiaoqiong Gu[7], Li Zhang[7], Rui Sun[8], Xunsha Sun[9], Jingyao Li[1], Jinxin Ou[1], Ting Xu[1], Xueying Huang[1], Yange Cao[1], Xiaohong Ruby Xu[4,5], Danielle Karakas[4,5], June Li[4,5,6], Heyu Ni[4,5,6,10], Qing Zhang[1,3]

Supplementary Figures

Supplementary Figure S1
Supplementary Figure S2
Supplementary Figure S3
Supplementary Figure S4
Supplementary Figure S5
Supplementary Figure S6

Supplementary Tables

Supplementary Table S1
Supplementary Table S2
Supplementary Table S3
Supplementary Table S5
Supplementary Table S6

Supplementary materials and methods

Supplementary Figures

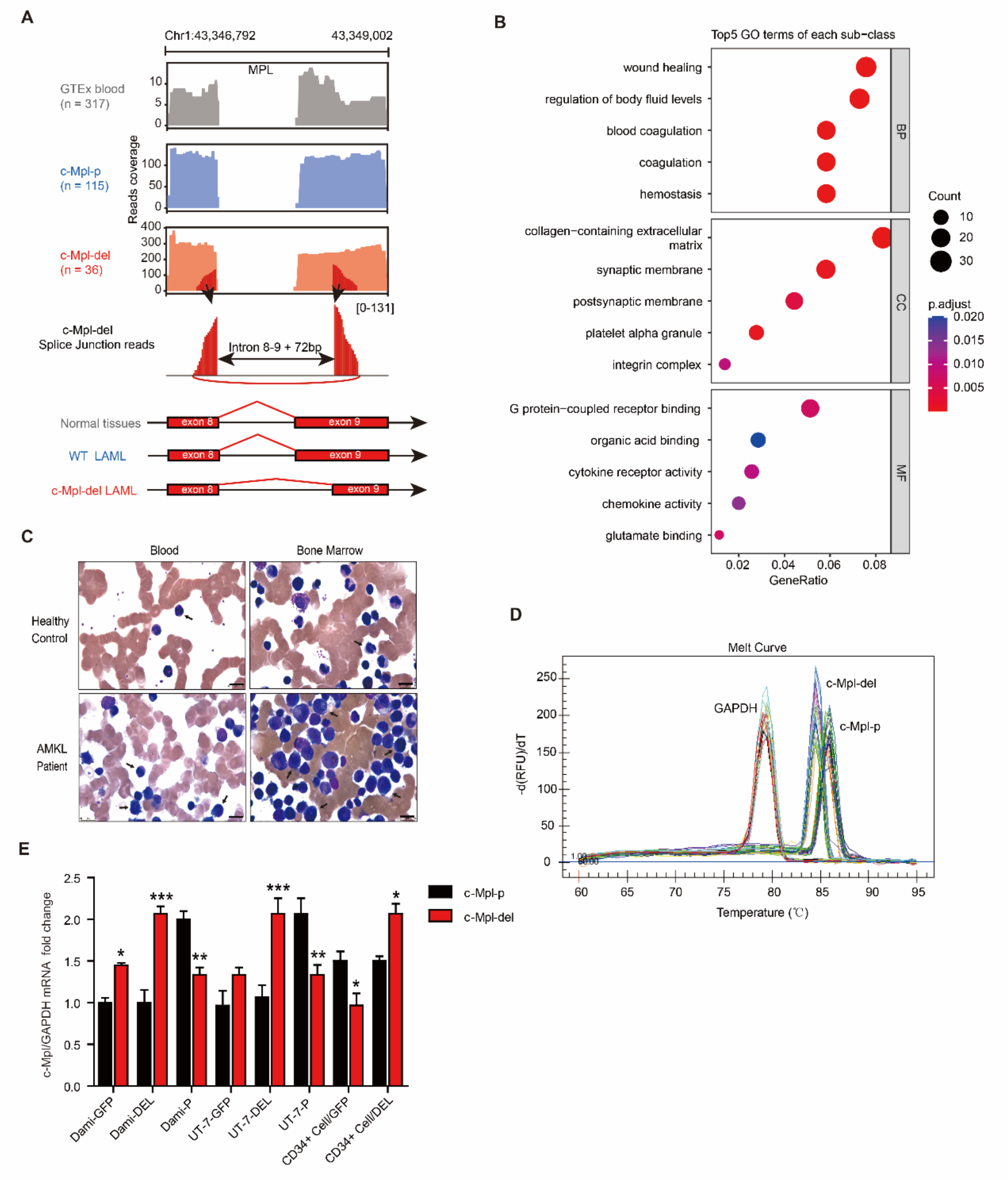

Supplementary Figure S1. Identification and characterization of c-Mpl-del expression

(**A**) Schematic approach to distinguish the c-Mpl-p and c-Mpl-del groups in TCGA-LAML and GTEx-blood, the splice junction reads in each bam file were extracted by

SAM tools. **(B)** Enrichment analysis of gene ontology (GO) terms and pathways in c-Mpl-del expressed AML patients from TCGA data set. BP, biological process; CC, cellular component; MF, molecular function. (**C**) Representative Wright-Giemsa staining depicting blast hypercellularity bone marrow smears in AMKL patients, arrows point to proliferative blasts (Scale bar 10 μm). (**D**) Melting curves analysis demonstrates presence of c-Mpl-p and c-Mpl-del isoform transcripts and reference gene GAPDH. **(E)** Quantitative PCR analysis of c-Mpl-p and c-Mpl-del in AMKL stably transfected cell lines and human primary CD34+ cells. Data represents the mean ± SD (n=3; $^{*}p<0.05$; $^{**}p<0.01$; $^{***}p<0.001$).

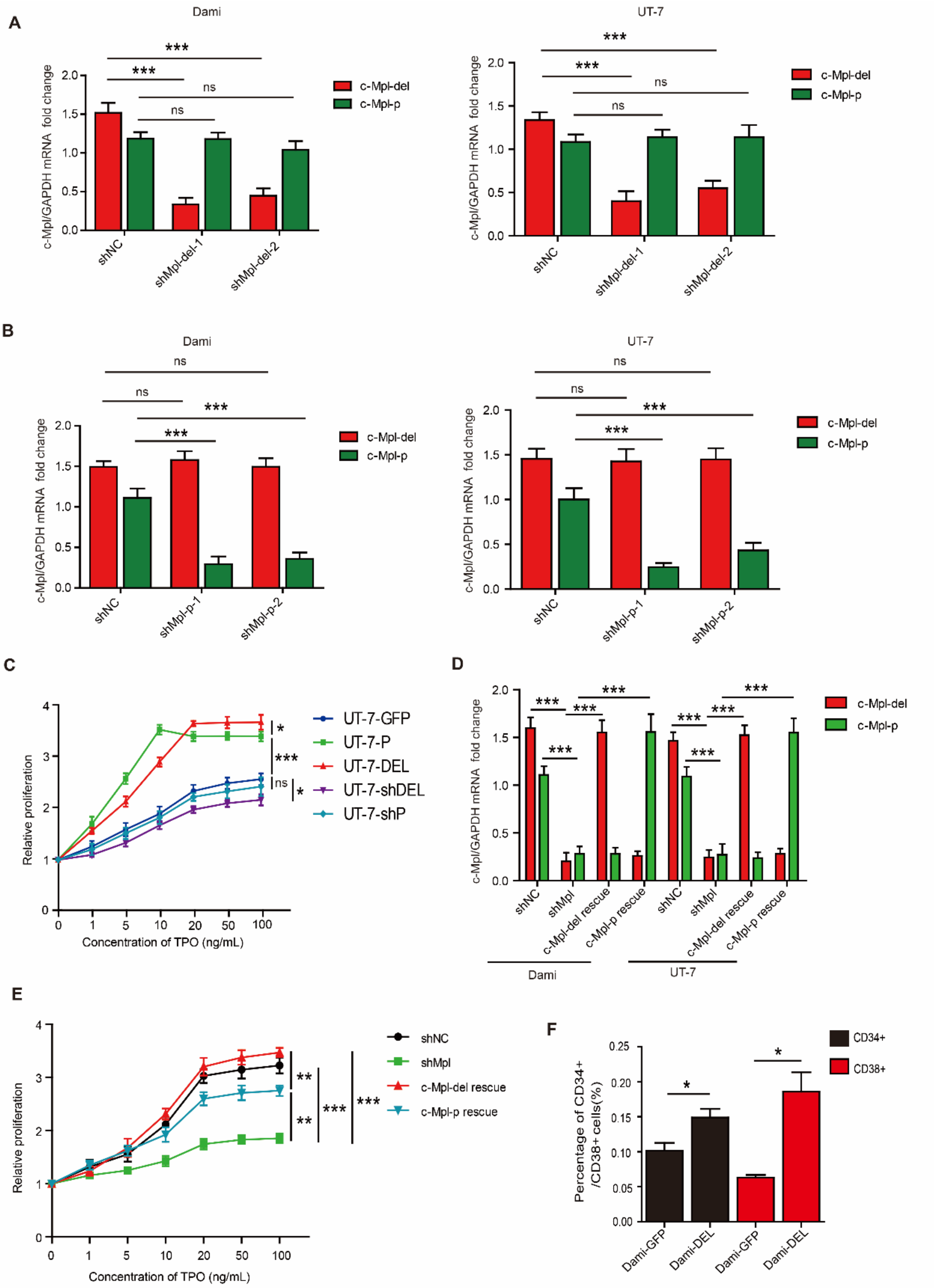

Supplementary Figure S2. c-Mpl-del enhances AMKL proliferation in response to TPO

(**A**) qRT-PCR showing the knockdown efficiency of the shRNAs targeting c-Mpl-del

in Dami and UT-7 cells. (**B**) qRT-PCR showing the knockdown efficiency of the shRNAs targeting c-Mpl-p in Dami and UT-7 cells. (**C**) Cell proliferation of c-Mpl-del (UT-7-DEL), c-Mpl-p (UT-7-P), control GFP (UT-7-GFP), c-Mpl-p shRNA (UT-7-shP), or c-Mpl-del shRNA (UT-7-shDEL) transduced UT-7 cells 48 hours following stimulation with various TPO concentrations as measured by CCK-8 cell proliferation assay. **(D)** qRT-PCR showing the knockdown efficiency of the shRNAs targeting c-Mpl and the amount of rescue of c-Mpl-del and c-Mpl-p expression in Dami and UT-7 cells. (**E**) Rescue assay showing proliferative activity of c-Mpl-del and c-Mpl-p rescue in UT-7 cells following knockdown of the endogenously expressed c-Mpl. c-Mpl-del or c-Mpl-p expressing lentivirus were used to transfect and rescue the expression of c-Mpl-del or c-Mpl-p. (**F**) The surface expression of CD34 and CD38 was assessed using flow cytometry after staining with respective APC- and PE-conjugated antibodies in Dami-GFP and Dami-DEL cells. Data represents the mean ± SD (n=3; $^{*}p<0.05$; $^{**}p<0.01$; $^{***}p<0.001$).

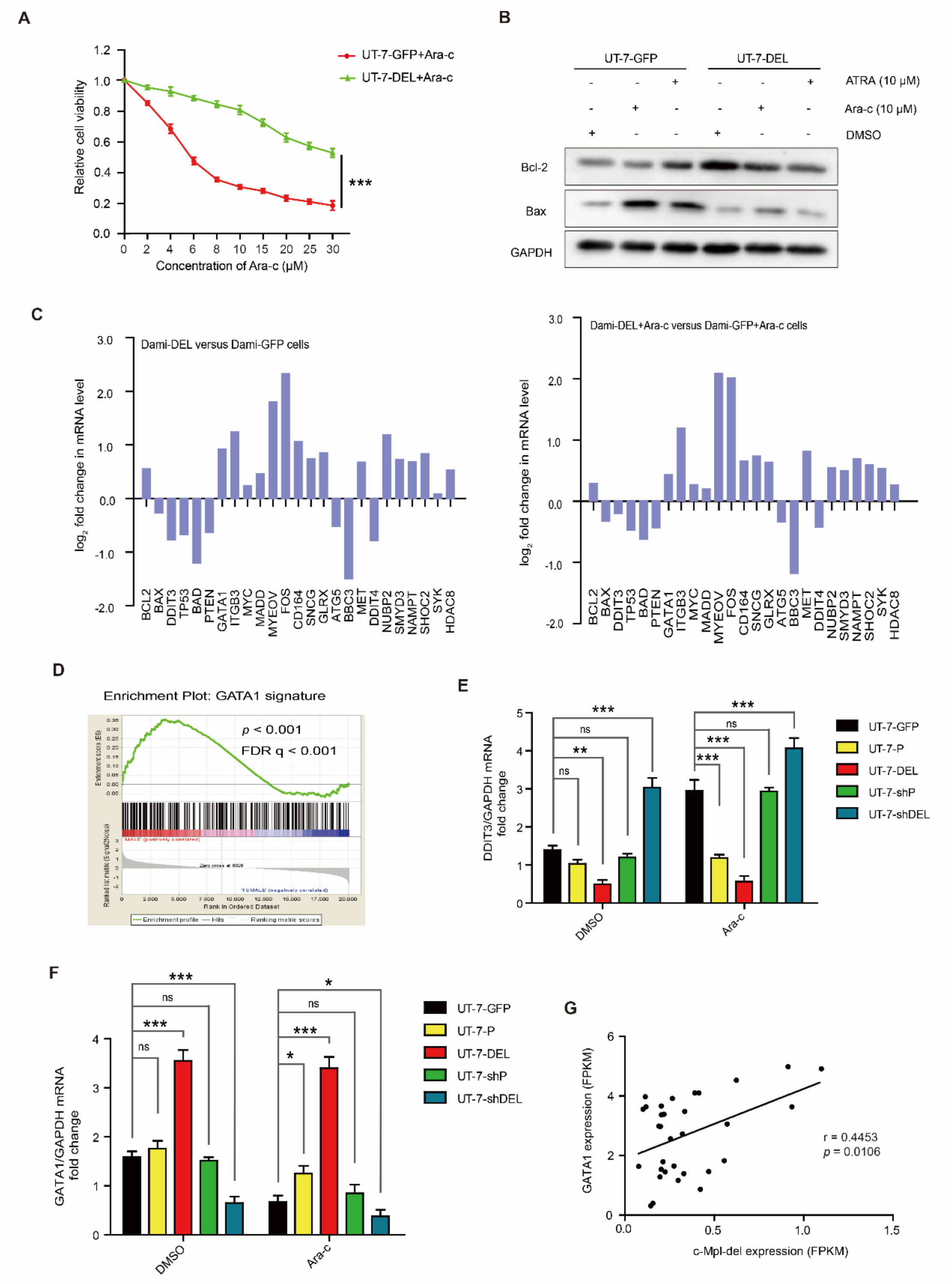

Supplementary Figure S3. c-Mpl-del overexpression promotes chemotherapeutic drug resistance

(A-B) Cell viabilities of differentially transfected UT-7 cells at 48 h post 20 ng/mL

TPO with indicated concentrations of Ara-c or ATRA treatment was measured using (**A**) CCK-8 viability assay and (**B**) Immunoblot analysis of Bcl-2 and Bax expression. All experiments were repeated three times. **(C)** Increased c-Mpl-del expression in Dami cells modulated the expression of a large cohort of survival genes in response to Ara-c stimuli in the presence of TPO. (**D**) GSEA indicated significant proliferation-related gene enrichment classified by GATA1 signature in Dami-DEL cells. (**E**) quantitative PCR analysis of DDIT3 expression in UT-7-GFP, UT-7-P, UT-7-DEL, UT-7-shP, and UT-7-shDEL cells treated with or without Ara-c. (**F**) quantitative PCR analysis of GATA1 expression in UT-7-GFP, UT-7-P, UT-7-DEL, UT-7-shP, and UT-7-shDEL cells treated with or without Ara-c. (**G**) Pearson correlation analysis of GATA1 and c-Mpl-del expression in c-Mpl-del expressed AML patients. The expression of c-Mpl-del is calculated according to the percentage of c-Mpl-del in total c-Mpl. Data represents the mean ± SD (n=3; $^{*}p<0.05$; $^{**}p<0.01$; $^{***}p<0.001$).

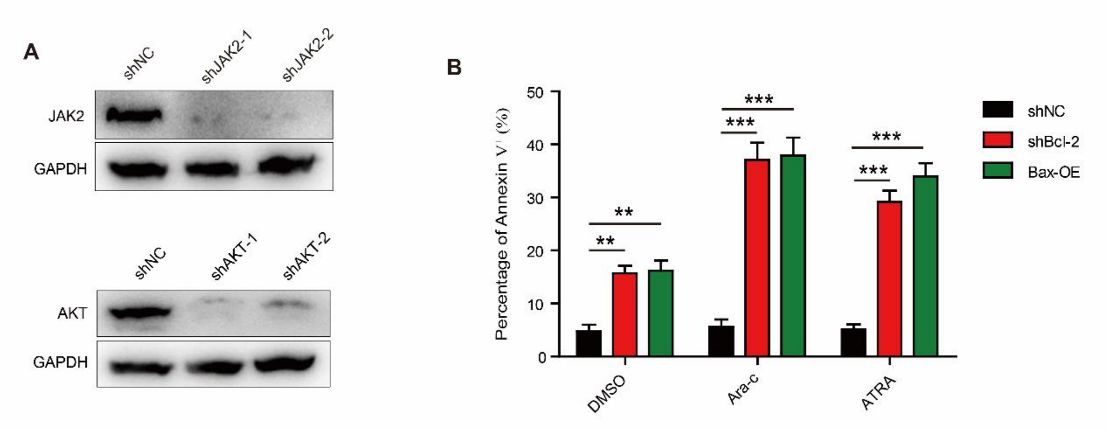

Supplementary Figure S4. Bcl-2 knockdown and Bax overexpression abolished c-Mpl-del-mediated anti-apoptotic responses in Dami cells.

(**A**) Western blot showing the knockdown efficiency of the shRNAs targeting JAK2 and AKT in Dami-DEL cells. (**B**) Flow cytometric Annexin V binding assay analysis of apoptosis in Dami-DEL cells following Bcl-2 knockdown and Bax overexpression in presence of Ara-c and ATRA. Data represent the mean ± SD (n=3; $^{**}p$<0.01; $^{***}p$<0.001).

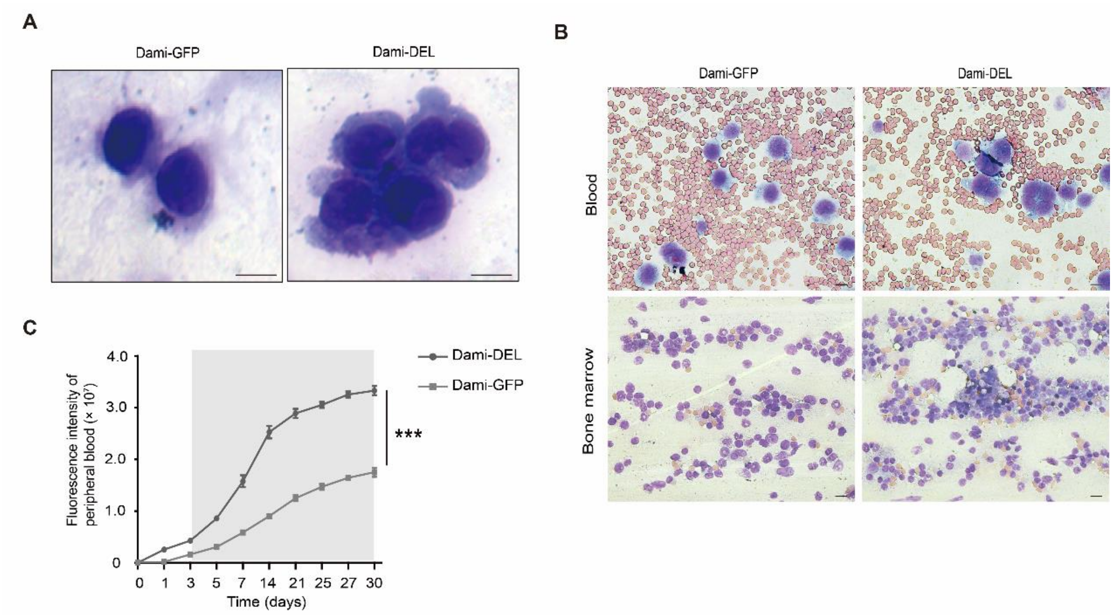

Supplementary Figure S5. c-Mpl-del promotes AMKL cell growth in NOD/SCID mice

(A) Wright-Giemsa staining was performed to evaluate morphological changes in AMKL ascitic cells. **(B)** Wright-Giemsa staining showing typical morphology of bone marrow and blood smears in from mice intravenously (IV) injected with Dami-DEL cells or Dami-GFP cells (Scale bar 10 μm). **(C)** Fluorescence positive AMKL cells in the peripheral blood at the indicated times after IV injection was detected via firefly luciferase substrate with a microplate reader. Data represents the mean ± SD (n=3; $^{***}p$<0.001).

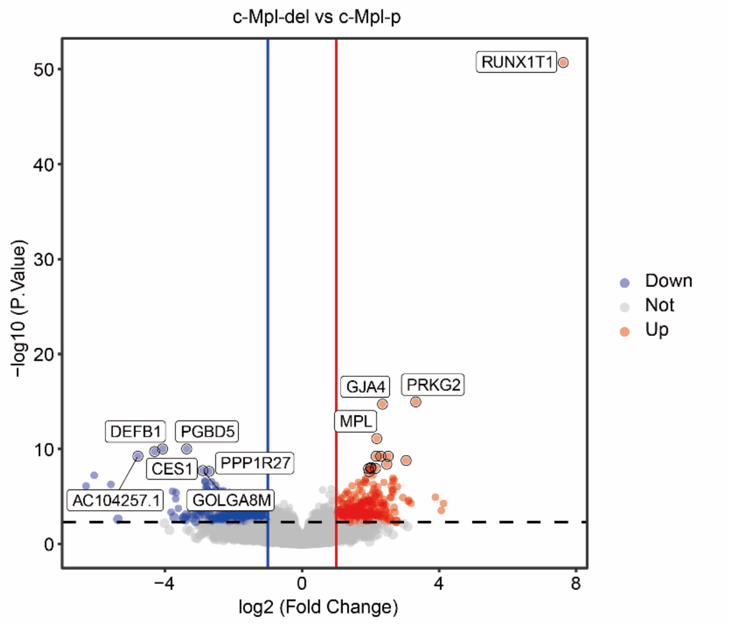

Supplementary Figure S6. Differential expression analysis revealing the differentially expressed genes between c-Mpl-del-expressed AML patients and without c-Mpl-del expressed AML patients.

Supplementary Tables

Supplementary Table S1. Pathway analysis of genes regulated by c-Mpl-del

Category term	Genes count	%	*P*-Value	Benjamini
PI3K-Akt signaling pathway	14	1.96	0.000105605	0.025456475
Rap1 signaling pathway	17	3.1	0.000501891	0.060491071
Cytokine-cytokine receptor interaction	18	3.61	0.001075525	0.079363787
p53 signaling pathway	8	0.97	0.001316954	0.079363787
MAPK signaling pathway	13	2.34	0.00200128	0.096482784
TNF signaling pathway	10	1.58	0.002635995	0.105902259
Cell cycle	9	1.36	0.003132517	0.107871624
Pathways in cancer	6	0.72	0.005020055	0.137993715
Staphylococcus aureus infection	7	0.96	0.005152167	0.137993715
Proteoglycans in cancer	14	3.07	0.008274715	0.199464185
Primary immunodeficiency	5	0.63	0.011971169	0.262334704
HTLV-I infection	16	3.95	0.014337911	0.288015938
Salmonella infection	7	1.21	0.017543404	0.306269642
Malaria	21	5.8	0.017787713	0.306269642
Histidine metabolism	3	0.33	0.036401845	0.511605901
Jak-STAT signaling pathway	14	3.73	0.037333866	0.511605901
Renin-angiotensin system	3	0.35	0.040451385	0.511605901
Basal cell carcinoma	5	0.89	0.046853995	0.511605901
Signaling pathways regulating pluripotency of stem cells	9	2.17	0.051759409	0.511605901
Chagas disease (American trypanosomiasis)	7	1.53	0.053151043	0.511605901

Supplementary Table S2. Pathway analysis of genes regulated by c-Mpl-del upon Ara-c treatment

Category term	Genes count	%	*P*-Value	Benjamini
Cell cycle	14	1.36	6.07E-05	0.014886305
Cytokine-cytokine receptor interaction	24	3.61	0.000267253	0.032773675
PI3K-Akt signaling pathway	8	0.72	0.001434757	0.117297658
MAPK signaling pathway	16	2.34	0.002031275	0.118142107
Arginine biosynthesis	5	0.32	0.002408476	0.118142107
Phospholipase D signaling pathway	15	2.22	0.003234262	0.132207537
Nitrogen metabolism	4	0.24	0.004920882	0.161798608
Arachidonic acid metabolism	8	0.92	0.006550633	0.161798608
Rap1 signaling pathway	18	3.1	0.006575729	0.161798608
Pathways in cancer	9	1.11	0.006596939	0.161798608
Transcriptional misregulation in cancer	15	2.53	0.010505453	0.234236423
Acute myeloid leukemia	7	0.86	0.015565525	0.318137484
Fructose and mannose metabolism	5	0.51	0.019217309	0.327482518
Fc epsilon RI signaling pathway	7	0.9	0.019791193	0.327482518
Jak-STAT signaling pathway	4	0.35	0.020028437	0.327482518
Glycerophospholipid metabolism	9	1.36	0.023107486	0.3521976
Endocytosis	19	3.84	0.025695338	0.3521976
B cell receptor signaling pathway	8	1.17	0.025847978	0.3521976
Phenylalanine metabolism	3	0.24	0.033279523	0.392295062
Hematopoietic cell lineage	9	1.47	0.036019298	0.392295062

Supplementary Table S3. AMKL patient characteristics

Characteristics	Age at diagnosis, y	Sex	BM blast (%)	Platelet count (10^9/L)	WBC count (10^9/L)	Hgb level (g/L)
UPN 1	1	Female	71.5	129	22.4	83
UPN 2	2	Male	69.5	91	6.2	140
UPN 3	2	Female	69.5	38	13.5	85
UPN 4	3	Male	88.5	40	4.2	76
UPN 5	3	Female	39.0	119	4.7	93
UPN 6	13	Male	34.5	77	3.7	75
UPN 7	67	Male	59.5	125	24.4	42
UPN 8	74	Male	46.5	356	2.3	59
UPN 9	76	Male	59.0	13	3.6	102
Average value	/	/	59.7	109	9.4	84

Abbreviations: AMKL, acute megakaryoblastic leukemia; UPN, unique patient number.

Supplementary Table S5. Sequences of shRNAs

shRNA	Sense sequence (5’-3’)	Reference
shMpl-del-1	AGGACTGGAAGCTGCGCGCCA	/
shMpl-del-2	ACTGGAAGCTGCGCGCCAGGCT	/
shMpl-p-1	GCGATCTCGCTACCGTTTACA	/
shMpl-p-2	GCTACCGTTTACAGCTGCGCG	/
shMpl	GCCCAAGAGACCTGTTATCAA	/
shJAK2-1	GCTTTGTCTTTCGTGTCATTA	Besancenot et al (1)
shJAK2-2	GAGTATGTGTCTGTGGAGA	Besancenot et al (1)
shAKT-1	GCTACTTCCTCCTCAAGAATG	Hou et al (2)
shAKT-2	GCTGGAGAACCTCATGCTG	Hou et al (2)
shBcl-2-1	GGGATCGTTGCCTTATGCATTTGTT	Chan e al (3)
shBcl-2-2	TAGCTGGATTATAACTCCTCTTCTT	Chan e al (3)
shNC	CGGCAGCTAGCGACGCCAT	Besancenot et al (1)

Supplementary Table S6. Information of antibodies used in this study

Antibody	Corporation	Cat.No.	Antibody	Corporation	Cat.No.
anti-Mpl	Santa Cruz	sc-377417	anti-phospho-ERK1/2	Abcam	ab76299
anti-Mpl	MilliporeSigma	SAB5300050	anti-ERK1/2	Abcam	ab54230
anti-Mpl	Abcam	ab232755	anti-phospho-AKT	Abcam	ab192623
anti-phospho-JAK2	Abcam	ab32101	anti-AKT	Abcam	ab179463
anti-JAK2	Abcam	ab108596	anti-DDIT3	CST	#2895
anti-phospho-STAT5	CST	#4322	anti-Ki-67	Abcam	ab92742
anti-STAT5	CST	#25656	anti-PTEN	Abcam	ab32199
anti-Bcl-2	CST	#15071	anti-HA	CST	#3724
anti-Bax	CST	#5023	anti-Myc	CST	#5605
anti-TPO	Santa Cruz	sc-366265	anti-β-actin	Abcam	ab6276
Anti-GATA1	Novus	NBP1-47492	anti-GAPDH	Abcam	ab8245

Supplementary materials and methods

RNA fluorescence in situ hybridization (FISH)

FISH analysis was performed in HEK293T, K562, NB4, Meg-01, UT-7, and Dami cells using the Fluorescent in Situ Hybridization Kit (RiboBi, China) as previously described (4). Cy3-labeled c-Mpl-del probes specific for the 72 bp deletion region crossing exon 8 and exon 9 was synthesized by RiboBio (RiboBi, China). Briefly, cells were washed with PBS and fixed in 4% formaldehyde for 10 min at room temperature, followed by permeabilization with PBS containing 0.5% Triton X-100 on ice for 5 min. Cells were then blocked in the pre-hybridization buffer for 30 min at 37℃. Hybridization was carried out using the c-Mpl-del FISH Probe Mix (RiboBio, China) at 37℃ for overnight avoiding light. After washed with the 4×, 2×, and 1× SSC buffer, cell nuclei were stained with DAPI and imaged by LSM880 laser confocal microscope (Zeiss, Germany).

Human primary CD34+ cells isolation and transduction

Mononuclear cells from fresh umbilical cord blood were isolated by density gradient centrifugation according to the manufacturer's instructions of SepMate™ and Ficoll-Paque™ (STEMCELL, Canada). CD34+ cells were separated by MACS MS Column and MACS Separator (Miltenyi Biotec, Germany) according to the manufacturer's instructions. A total of 10^8 cells were incubated with 100 μL of FcR Blocking Reagent followed by 100 μL of CD34 MicroBeads incubation for 30 minutes at 4 °C for Magnetic labeling and magnetic separation with MS Columns. Primary CD34+

cells were transduced with vector lentivirus or c-Mpl-del-expressing lentivirus, followed by culture with differing TPO concentrations (1, 5, 10, 20, 50, or 100 ng/mL) for 48 h to analyze proliferation via CCK-8 assay (Dojindo, Japan) or analysis of expression of Ki-67, PTEN, Bcl-2 and Bax via Western blot.

RNA isolation and quantitative real-time PCR (qRT-PCR)

Total RNA was extracted from bone marrow and leukemic cells from AMKL patients using RNAiso Plus reagent (Takara, Japan) in accordance with the manufacturer's instructions. RNA was reverse transcribed into cDNA using the RT reagent Kit RR047A (Takara, Japan) for real-time PCR with a SYBR Premix ExTaq real-time PCR Kit (Takara, Japan). The c-Mpl-p primers were 5'-AAACTTGCACTGGAGGGAGA-3' and 5'-GCAGCTGTAAACGGTAGCGA-3'. The c-Mpl-del primers were 5'-CCAAACTTGCACTGGAGGGA-3' and 5'-TGGCGCGCAGCTTCCAGT-3'.

The DDIT3 and GATA1 primers were previously published (5, 6). Quantitative PCR was performed using a StepOne Plus Sequence Detection System (Applied Biosystems, USA). All gene expression levels were normalized to GAPDH.

TPO quantification

TPO concentrations were evaluated from plasma of AMKL patients or healthy donors and AMKL cell culture medium using human TPO enzyme-linked immunosorbent (ELISA) kit (Elabscience, China) according to the manufacturer's instructions. The absorbance was measured and the TPO concentration was calculated according to the

standard concentration curve.

Flow cytometry analysis of cell surface c-Mpl

Single-cell suspensions were prepared from bone marrow of healthy controls and AMKL patients as previously reported standard techniques (7). To evaluate the cell surface c-Mpl expression, a total of 100 μL cells (1×10^6) were incubated with primary mouse anti-human c-Mpl antibodies followed by FITC-conjugated IgG secondary antibodies. Mouse IgG was used as isotype control. Cells were then washed 3 times by centrifugation at 400g and analyzed on a FC500 flow cytometer (Beckman, Germany).

Cell proliferation and cell viability assays

Growth factor-starved cells were seeded into 96-well plates at a density of 3000 cells/well and treated with differing TPO concentrations of (0, 1, 5, 10, 20, 50, or 100 ng/mL). After 48 h, 10 µl of CCK-8 (Dojindo, Japan) was added to each well, and the cells were incubated for 5 h. Absorbance was measured at 450 nm using a nucleic acid protein analyzer (Bio-Tek Instruments, USA). For the cell viability assay, the growth factor-starved cells were seeded into 96-well plates at a density of 5×10^4/well in the presence of 20 ng/mL TPO and various concentrations of Ara-c or ATRA (0, 2, 4, 6, 8, 10, 15, 20, 25, or 30 μM). The results were detected via the same method used for the cell proliferation assay.

Colony formation assay

A total of 400 AMKL cells was added to 1.2 mL of methylcellulose media (R&D, USA). The vial was vortexed vigorously and allowed to stand until no bubbles appeared. Subsequently, 500 μL of the final cell mixture was seeded per well in a 24-well cell culture plate. The colony numbers and numbers of cells in single clones were counted and assessed for colony formation in the presence of 20 ng/mL TPO for 14 days at 37 ℃ and 5% CO_2 using a Leica DM6B microscope (Leica, Germany).

Cell cycle and apoptosis assays

Ascitic and cultured cells were collected from ascitic fluids obtained from transplanted mice or cultured AMKL cells, respectively, and treated with chemotherapeutic drugs. For the cell cycle analysis, a total of 1×10^5 AMKL cells were fixed, permeabilized, and stained with 7-AAD (Biolegend, USA) for 15 min at room temperature in the dark. Data were acquired by FC500 flow cytometry (Beckman, Germany), and the cell cycle profile was processed by FlowJo software. For the apoptosis analysis, a total of 1×10^5 cells were labeled with FITC-conjugated Annexin V and 7-AAD (Biolegend, USA) for 15 min at room temperature in the dark and then analyzed using an FC500 flow cytometer (Beckman, Germany).

Western blot and Co-IP

Cells were lysed with RIPA buffer on ice for 20 minutes, followed by centrifugation at 12000 rpm for 3 minutes to remove cell debris. Protein extracts were resolved via 10%

or 15% SDS-PAGE, transferred onto polyvinylidene fluoride membranes (Millipore, Germany), probed with the appropriate antibody overnight at 4 ℃ and followed by horseradish peroxidase-conjugated secondary antibodies incubation at room temperature for 1h. Membranes were visualized with an enhanced chemoluminescence detection system (Tanon, China). Detail information of antibodies were summarized in **Table S6**. For Co-IP, Whole-cell lysates were incubated successively with monoclonal antibodies for 4-6 hours and followed by capture with protein A/G agarose beads for 12 to 16 hours. Bound proteins were then washed in lysis buffer, resuspended in protein sample buffer, separated by SDS-PAGE, and detected by immunoblot.

Total RNA isolation and expression analysis

Dami-GFP, Dami-DEL, and Dami-P cells were treated with or without Ara-c (10 μM) in the presence of 20 ng/mL TPO for 48 h. Total RNA was extracted using TRIzol (Invitrogen, USA) according to the manufacturer's instructions. cDNA libraries were constructed following the High-Throughput Illumina Strand-Specific RNA Sequencing Library protocol. The library products were sequenced on an Illumina HiSeq™ 2500. RNA-library sequencing and transcript assembly were performed by Beijing Biomarker Technology, Inc. All usable reads that could be uniquely mapped to a gene were used to calculate the expression level. The numbers of gene reads were further measured by assessing the number of uniquely mapped reads per kilobase of exon region per million mappable reads (RPKM) as previously report (8), which is based on the Audic method

(9) for analyzing differential expression. All variants of the experiment were repeated three times and analyzed with their mean values. Significantly differential expressed genes in different samples were selected using edgeR with a threshold of FDR < 0.01 & Fold Change ≥ 2. All identified differentially expressed genes for each comparison group of different experimental conditions were summarized in **Table S7**. All the transcriptome raw data have been deposited under the GEO accession number GSE127762 and GSE145949.

Chromatin immunoprecipitation-quantitative PCR (ChIP-qPCR)

ChIP-qPCR was carried out as previously described (10). Briefly, cross-linking was performed in cell culture medium containing 1% formaldehyde and terminated after 5 min by addition of glycine at a final concentration of 0.125 M. Cells were harvested with lysis buffers in ChIP kit (BersinBio, Guangzhou, China) according to the manufacturer's instructions. Chromatin was sonicated with SCIENTZ-950E (Scientz Biotechnology, China) to generate 200 to 600 bp DNA fragments. Immunoprecipitation reactions were performed with antibodies against DDIT3 (CST, USA) or with normal IgG used as a negative control. After the reverse cross-linking and elution immunoprecipitated chromatin, purified DNA fragments were subjected to qPCR analysis. Primers used for ChIP-qPCR are as follows: Bcl-2 (forward: 5′-AATTTGGTGTGCTTTTCTGG-3′ reverse: 5′-GTGTGCAGTGGTGCGATCTC- 3′), Bax (forward: 5′-CCATGATTGGGCCACTGCAC-3′ reverse: 5′-

AGCTCTCCCCAGCGCAGAAG-3′), and PTEN (forward: 5′-CTTTGAGCCCTCCCAGGCC-3′ reverse: 5′-CAACCGTGGGAGAAGAGGC-3′). The $2^{-\Delta\Delta CT}$ method was used to calculate the percentage of DNA target fragments relative to the input control in the ChIP reaction.

Morphologic studies

Peripheral blood smears and bone marrow aspirate smears were made using 10 μl of whole blood or bone marrow obtained from AMKL patients and normal donors or mice transplanted with AMKL cells and then stained with Wright's stain (Thermo Fisher Scientific, USA). The morphological analyses of the Dami-GFP and Dami-DEL cells obtained from the ascitic fluids of transplanted mice were performed using Wright-Giemsa staining. For histopathological analyses, bone marrow, lung, liver and spleen tissues from untransplanted NOD/SCID mice and Dami-GFP- and Dami-DEL-transplanted NOD/SCID mice were fixed with 4% polyphosphate formaldehyde, embedded in paraffin, and cut into 5-μm sections. Histopathological changes were assessed using standard hematoxylin and eosin (H&E) staining. The megakaryocytes in the bone marrow and spleen were counted in 10 randomly chosen fields to measure infiltration by AMKL cells.

In vivo bioluminescence imaging

Bioluminescence image acquisition and analysis were performed in irradiated NOD/SCID mice inoculated with Dami-GFP-Luc or Dami-DEL-Luc cells using a

Xenogen IVIS 50 cooled CCD optical system (Xenogen, USA) to monitor tumor growth and infiltration. Firefly D-luciferin (Promega, USA) was diluted to a 15 mg/mL stock in phosphate-buffered saline (PBS) and filtered before use. Different groups of mice were injected intraperitoneally with 100 μL of D-luciferin (150 mg/kg body weight) before imaging analysis. Images were acquired at one-minute intervals until the maximum whole-body signal was detected. Bioluminescence imaging flux values were serially monitored over a 14-day period, with the initial scans acquired at 5 h after irradiation. During the study, blood cells were harvested from transplanted mice at the indicated times for ex vivo bioluminescence intensity analysis to monitor the growth of Dami-GFP-Luc or Dami-DEL-Luc cells.

References:

1. Besancenot R, Roos-Weil D, Tonetti C, Abdelouahab H, Lacout C, Pasquier F, et al. JAK2 and MPL protein levels determine TPO-induced megakaryocyte proliferation vs differentiation. Blood. 2014;124(13):2104-15.
2. Hou YQ, Yao Y, Bao YL, Song ZB, Yang C, Gao XL, et al. Juglanthraquinone C Induces Intracellular ROS Increase and Apoptosis by Activating the Akt/Foxo Signal Pathway in HCC Cells. Oxid Med Cell Longev. 2016;2016:4941623.
3. Chan SM, Thomas D, Corces-Zimmerman MR, Xavy S, Rastogi S, Hong WJ, et al. Isocitrate dehydrogenase 1 and 2 mutations induce BCL-2 dependence in acute myeloid leukemia. Nat Med. 2015;21(2):178-84.
4. Wang WT, Chen TQ, Zeng ZC, Pan Q, Huang W, Han C, et al. The lncRNA LAMP5-AS1 drives leukemia cell stemness by directly modulating DOT1L methyltransferase activity inMLLleukemia. Journal of Hematology & Oncology. 2020;13(1).
5. Wang M, Yang Q, Long J, Ding Y, Zou X, Liao G, et al. A comparative study of toxicity of TiO2, ZnO, and Ag nanoparticles to human aortic smooth-muscle cells. Int J Nanomedicine. 2018;13:8037-49.
6. Xu E, Ji Z, Jiang H, Lin T, Ma J, Zhou X. Hypoxia-Inducible Factor 1A Upregulates HMGN5 by Increasing the Expression of GATA1 and Plays a Role in Osteosarcoma Metastasis. Biomed Res Int. 2019;2019:5630124.

7. Linger RMA, DeRyckere D, Brandao L, Sawczyn KK, Jacobsen KM, Liang X, et al. Mer receptor tyrosine kinase is a novel therapeutic target in pediatric B-cell acute lymphoblastic leukemia (Retraction of vol 114, pg 2678, 2009). Blood. 2012;120(7):1533-.
8. Zeng CW, Chen ZH, Zhang XJ, Han BW, Lin KY, Li XJ, et al. MIR125B1 represses the degradation of the PML-RARA oncoprotein by an autophagy-lysosomal pathway in acute promyelocytic leukemia. Autophagy. 2014;10(10):1726-37.
9. Audic S, Claverie JM. The significance of digital gene expression profiles. Genome Res. 1997;7(10):986-95.
10. Wang XY, Zhang X, Wang TY, Jia YL, Xu DH, Yi DD. Shortened nuclear matrix attachment regions are sufficient for replication and maintenance of episomes in mammalian cells. Mol Biol Cell. 2019;30(22):2761-70.
