# Supplementary material for: c-Mpl-del, a c-Mpl alternative splicing isoform, promotes AMKL progression and chemoresistance
Source: Cell Death Dis. 2022 Oct 13;13(10):869. doi: 10.1038/s41419-022-05315-5 (PMC9561678; doi:10.1038/s41419-022-05315-5)

Figure 1F

c-Mpl

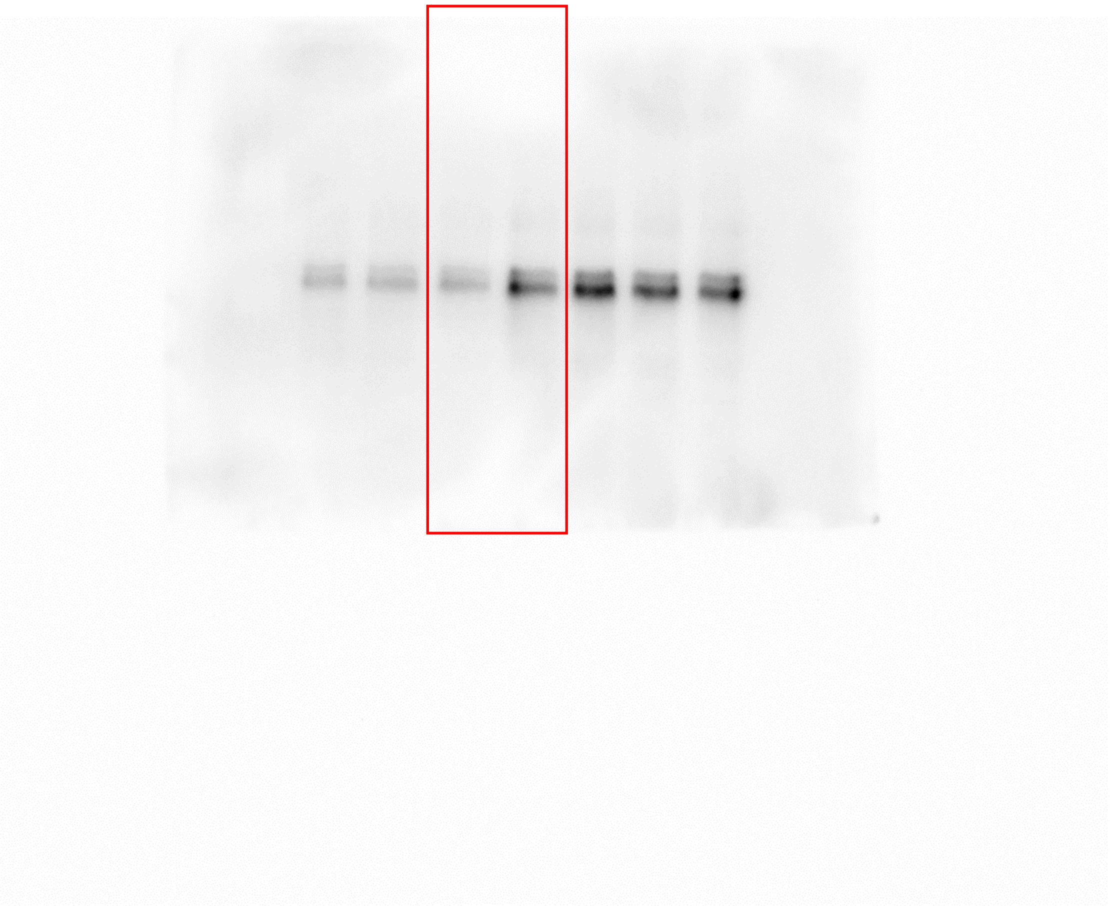

GAPDH

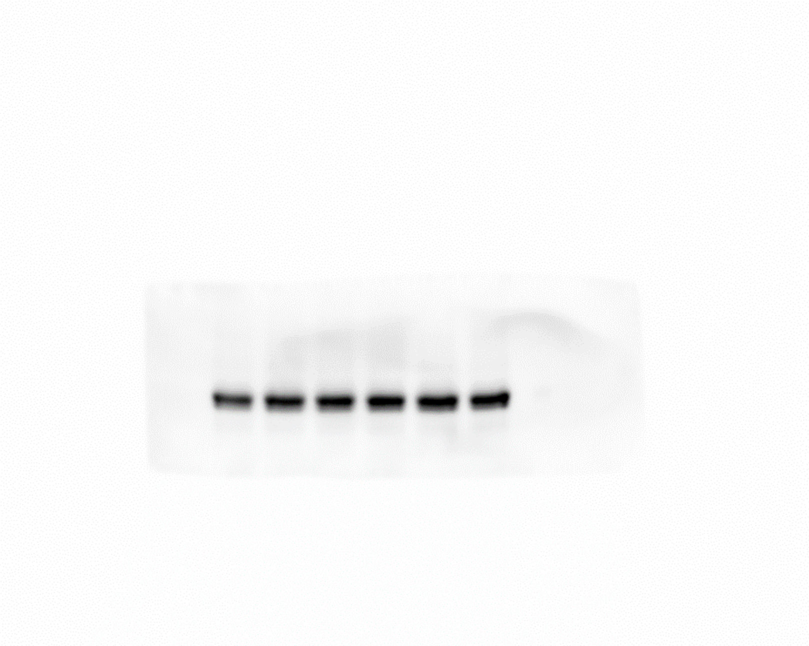

Figure 2G
Ki-67

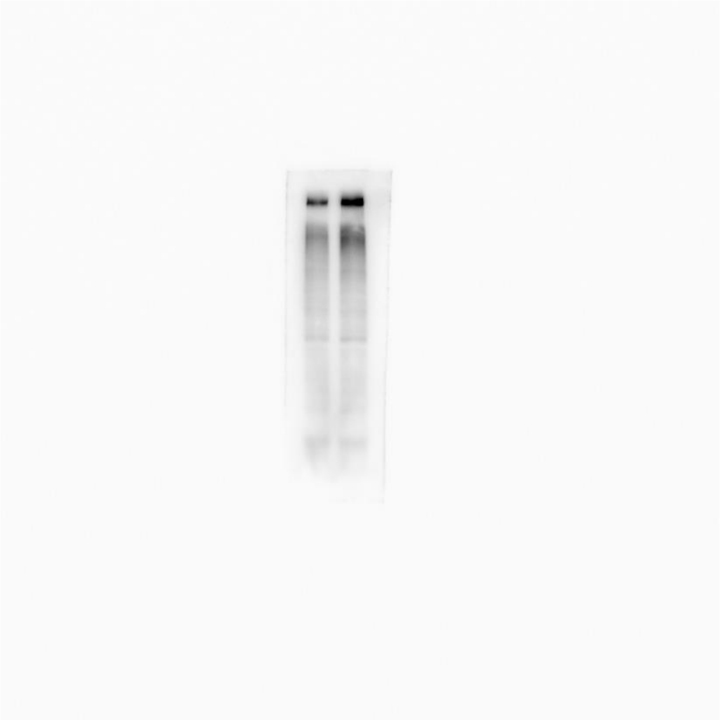

PTEN

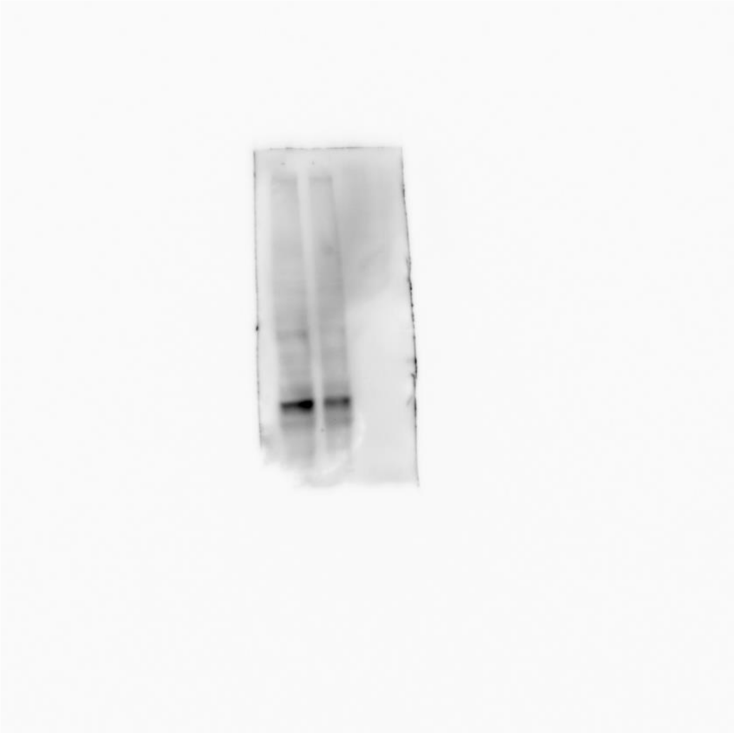

BCL-2

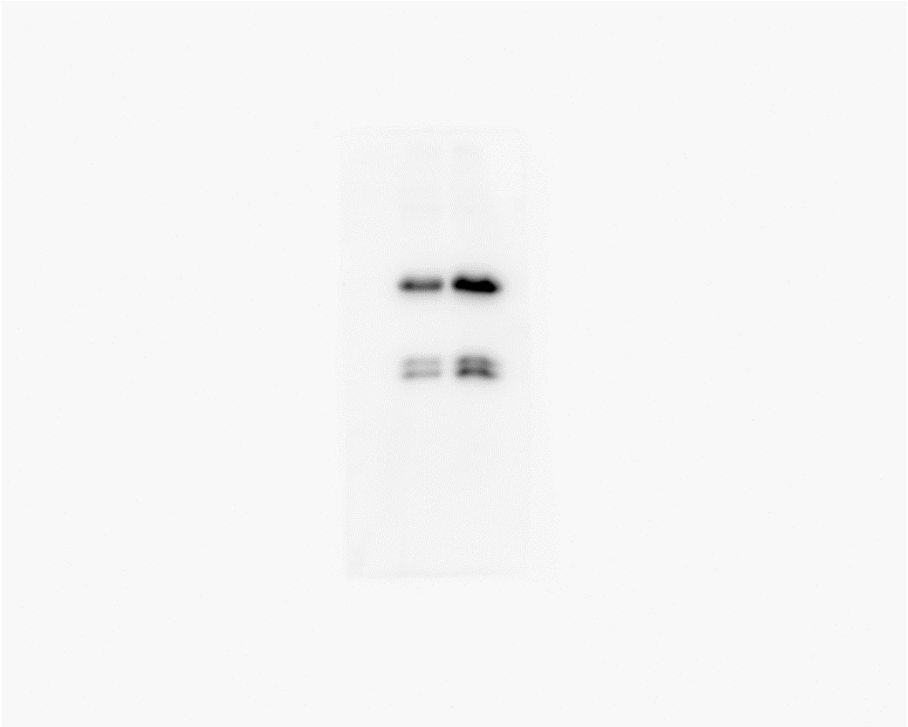

BAX

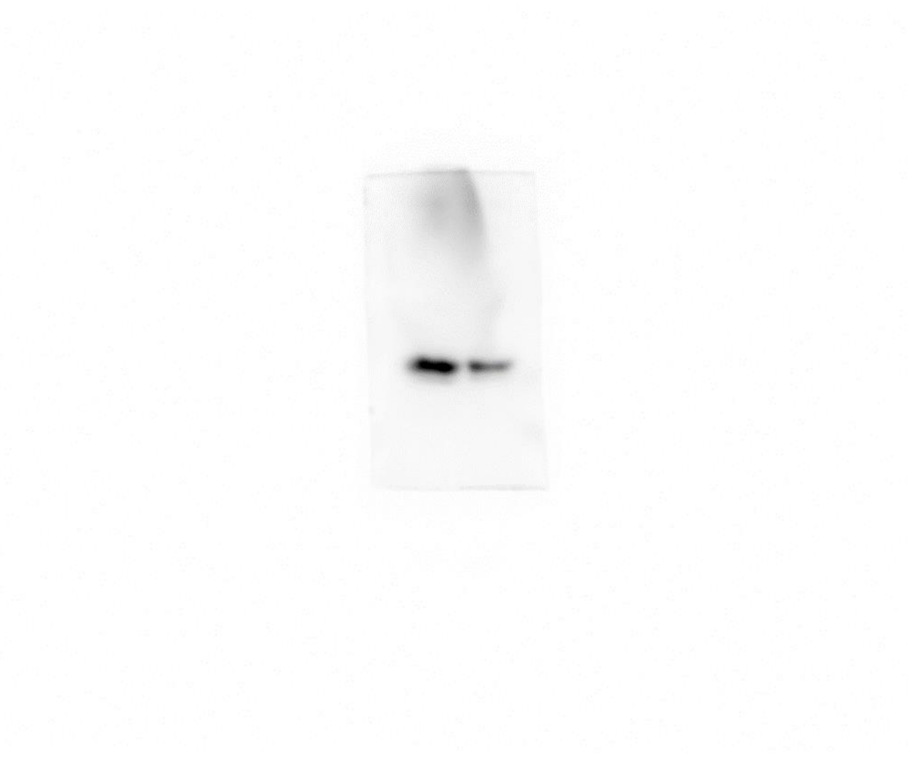

GAPDH

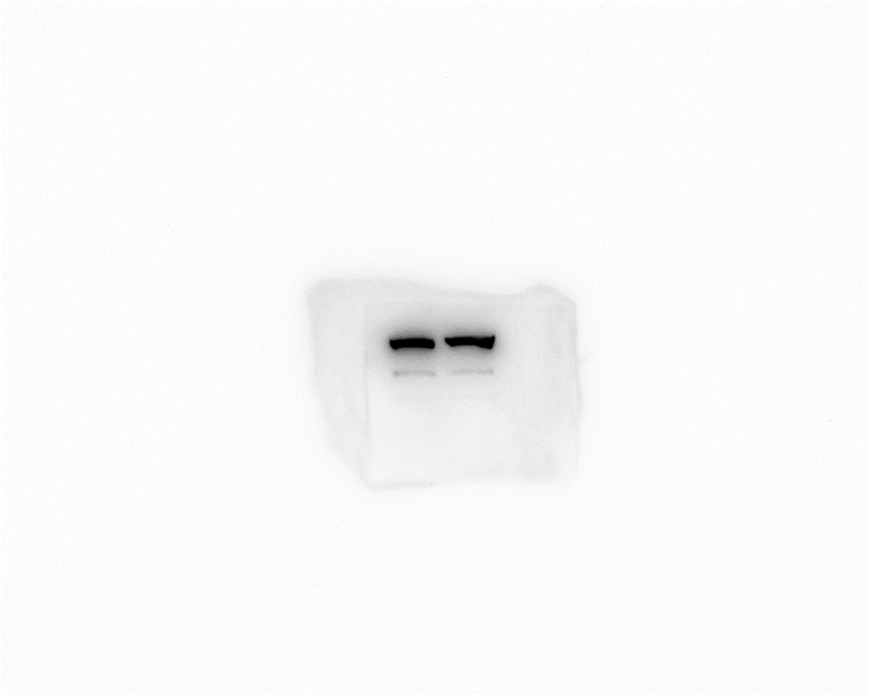

Figure 3C

BCL-2

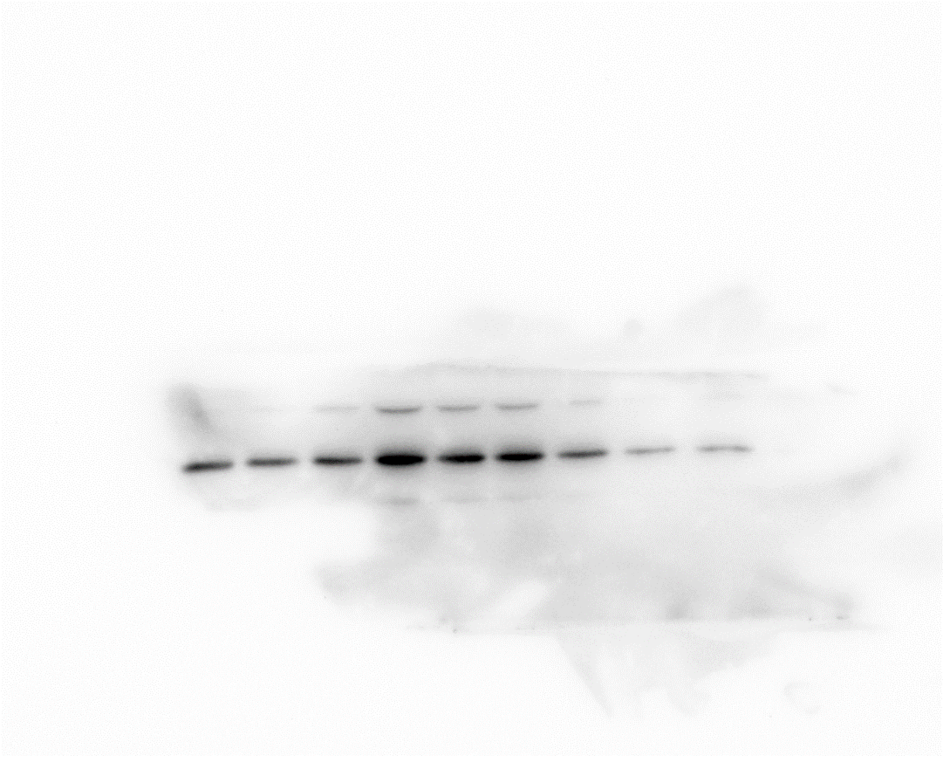

BAX

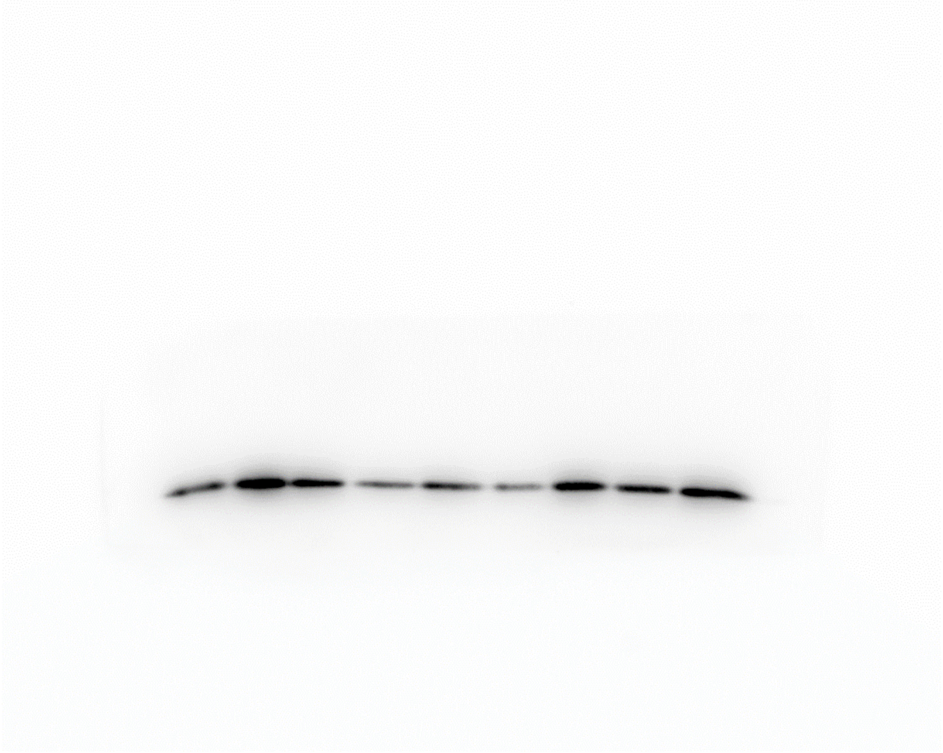

GAPDH

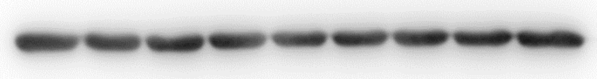

Figure 4A

Input: c-Mpl

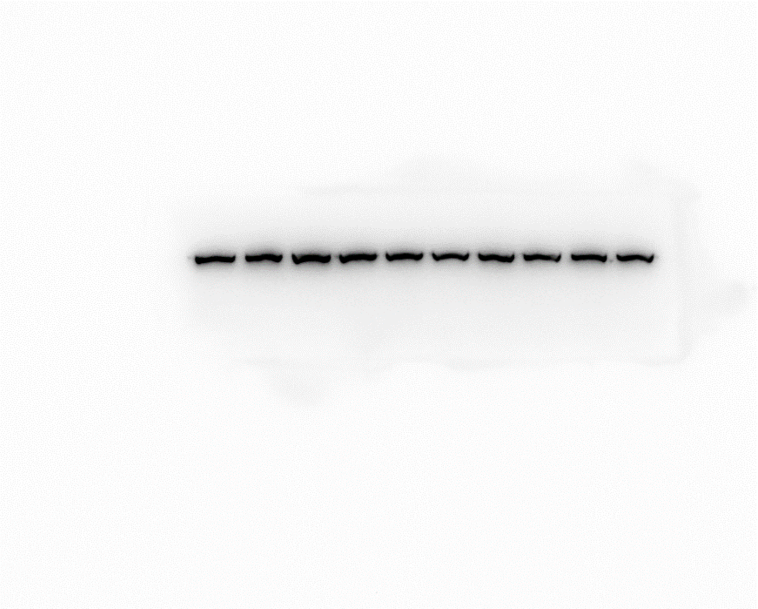

Input: P-JAK2

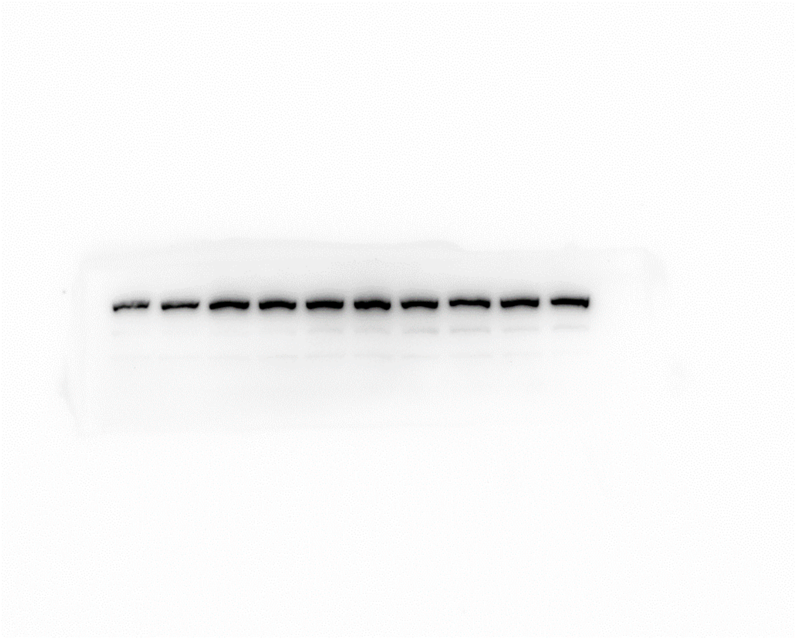

IP: c-MPL

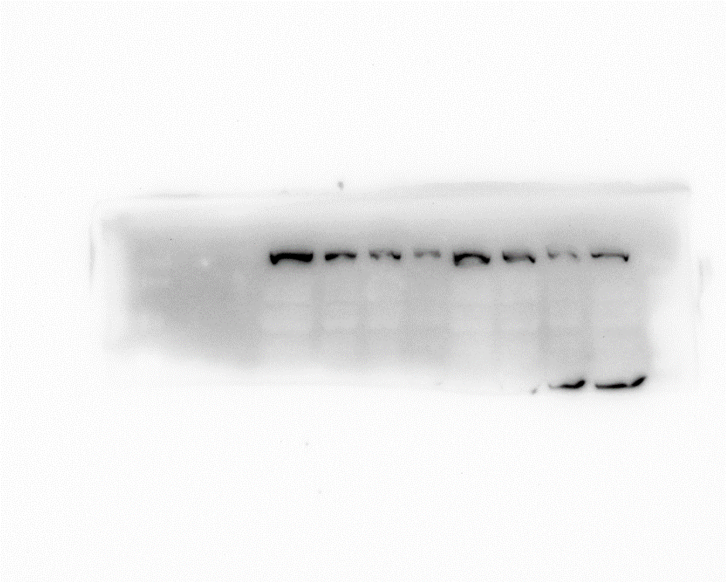

IP: P-JAK2

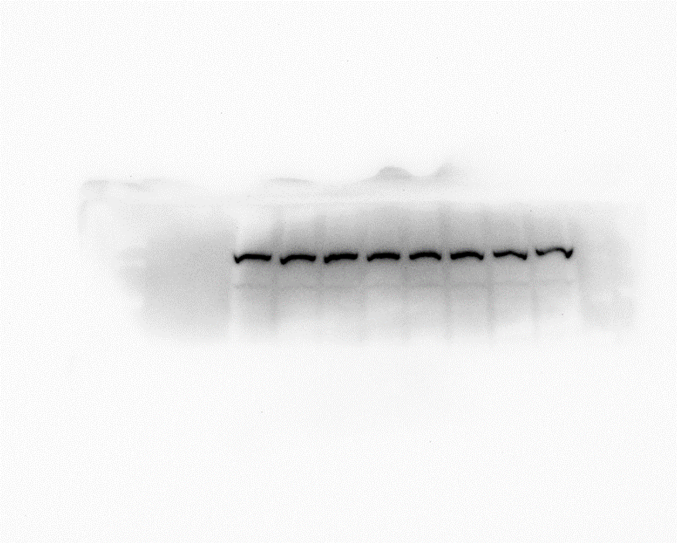

GAPDH

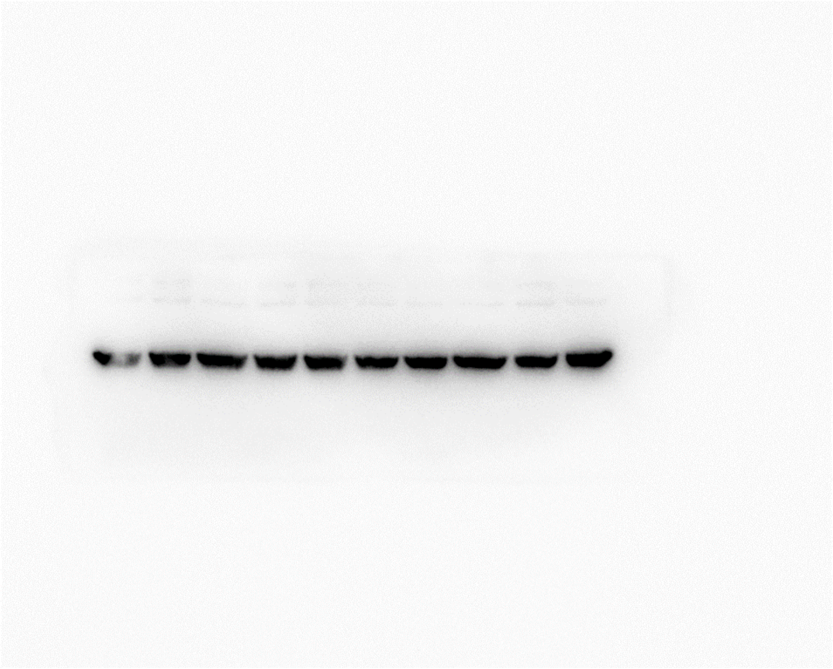

Figure 4B
P-JAK2

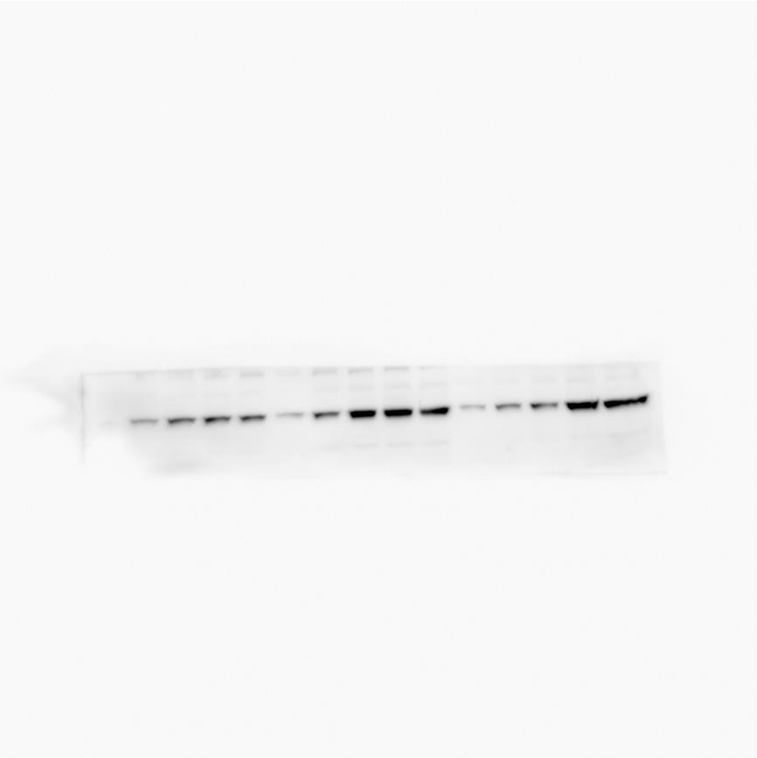

JAK2

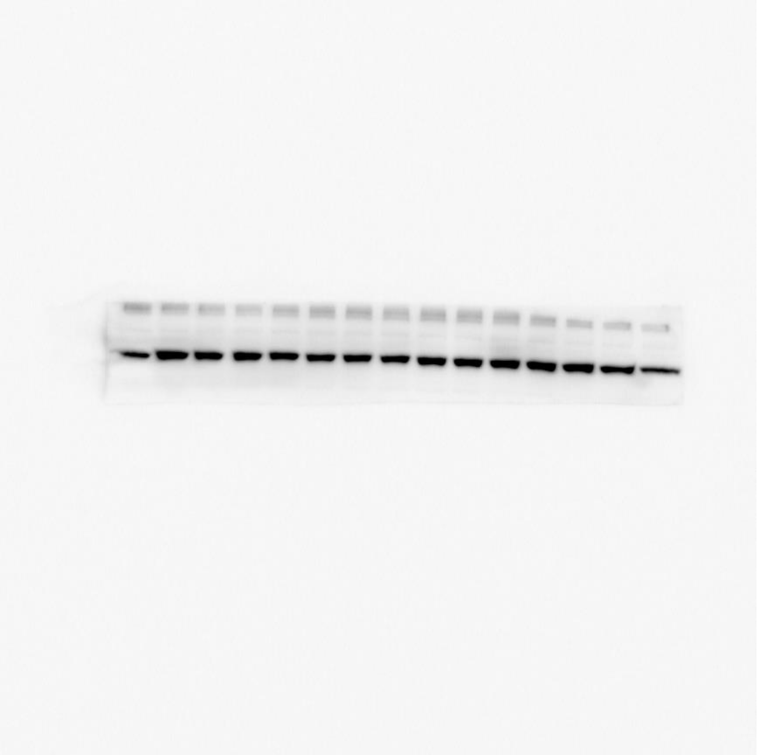

P-STAT5

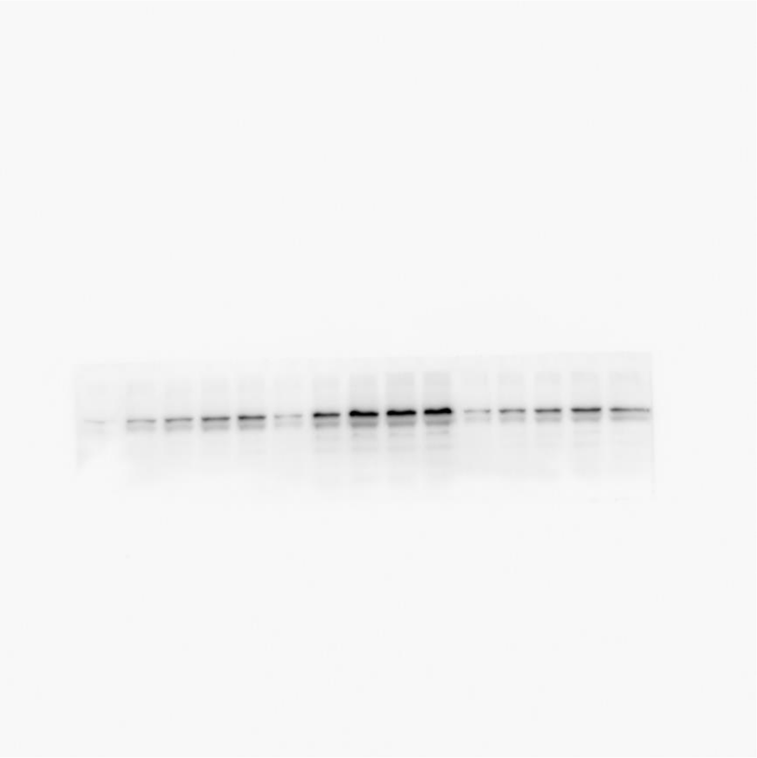

STAT5

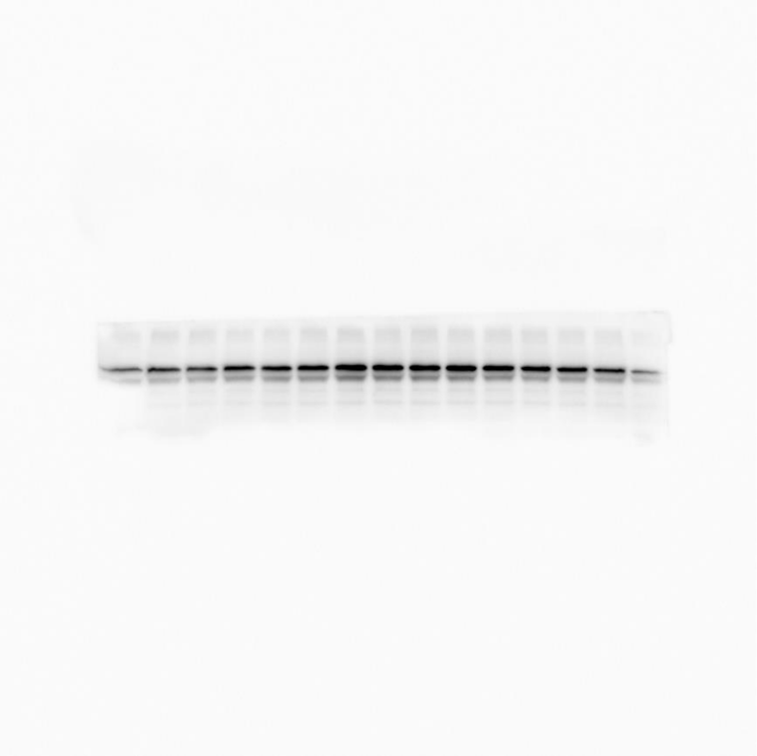

P-AKT

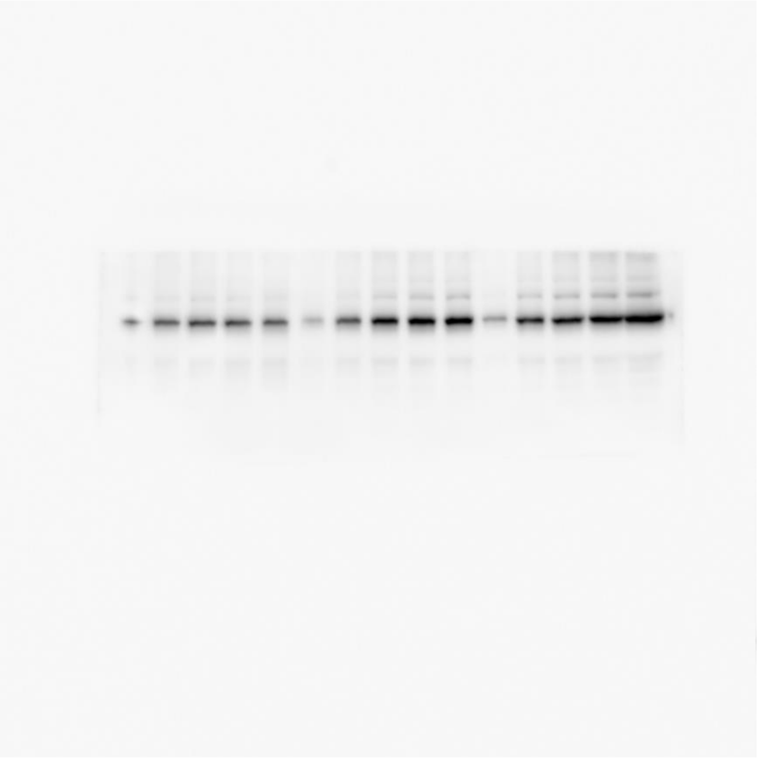

AKT

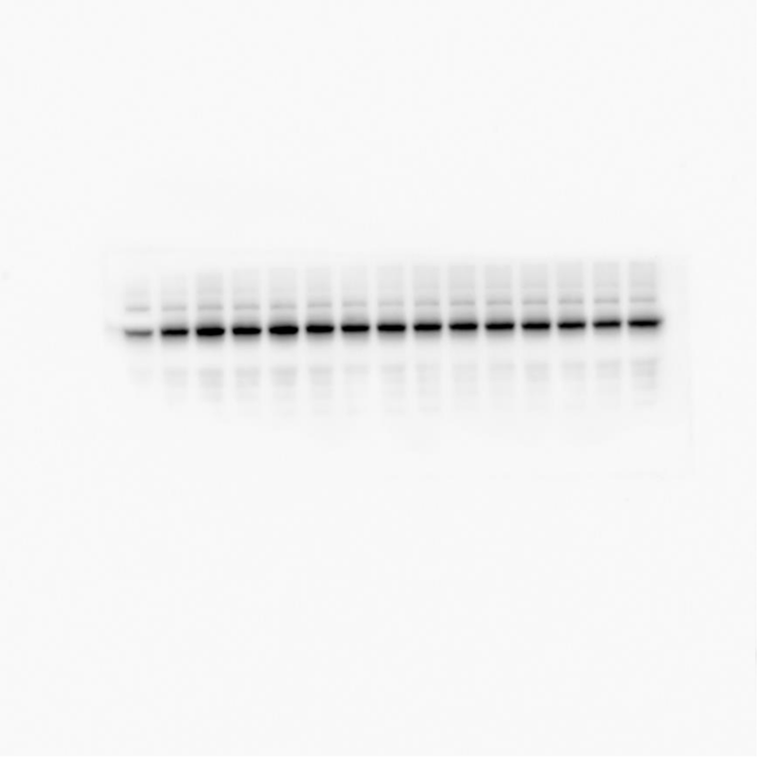

P-ERK1/2

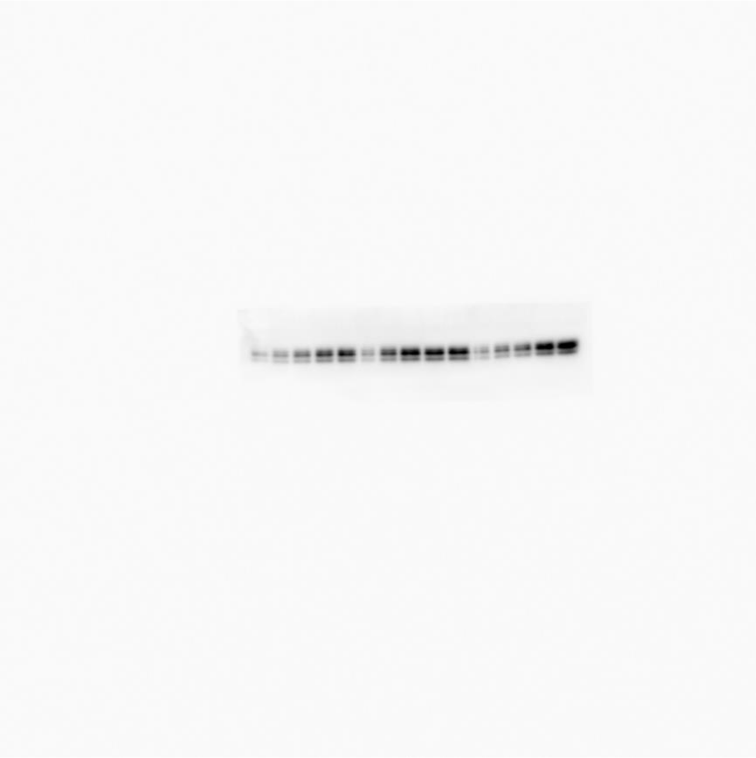

ERK1/2

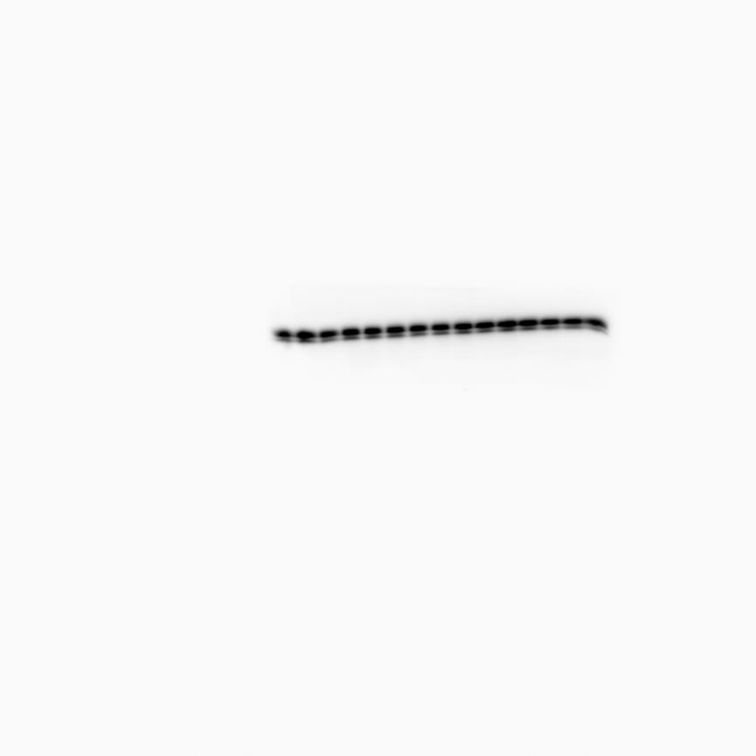

GAPDH

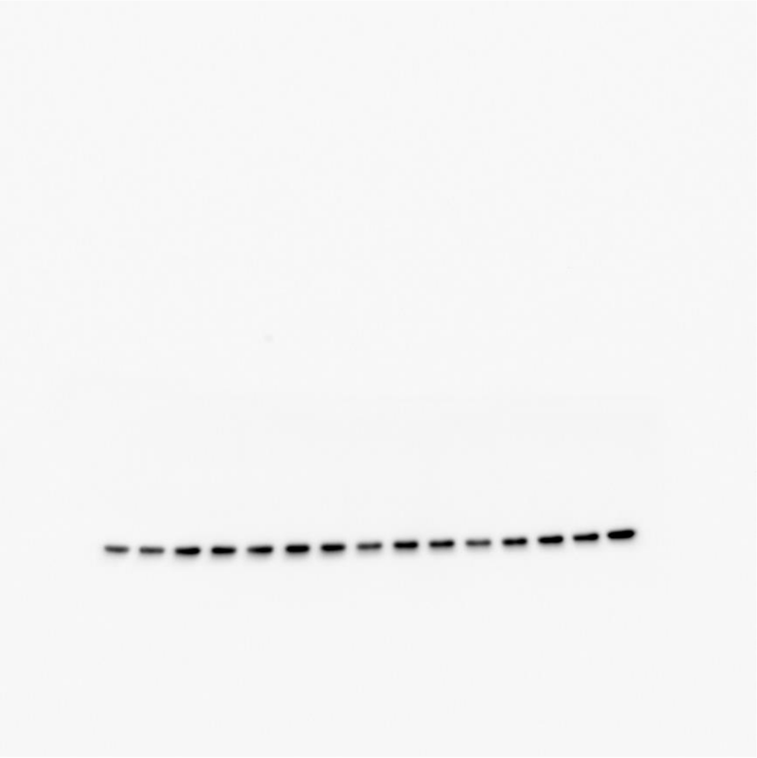

Figure 4D
BCL-2

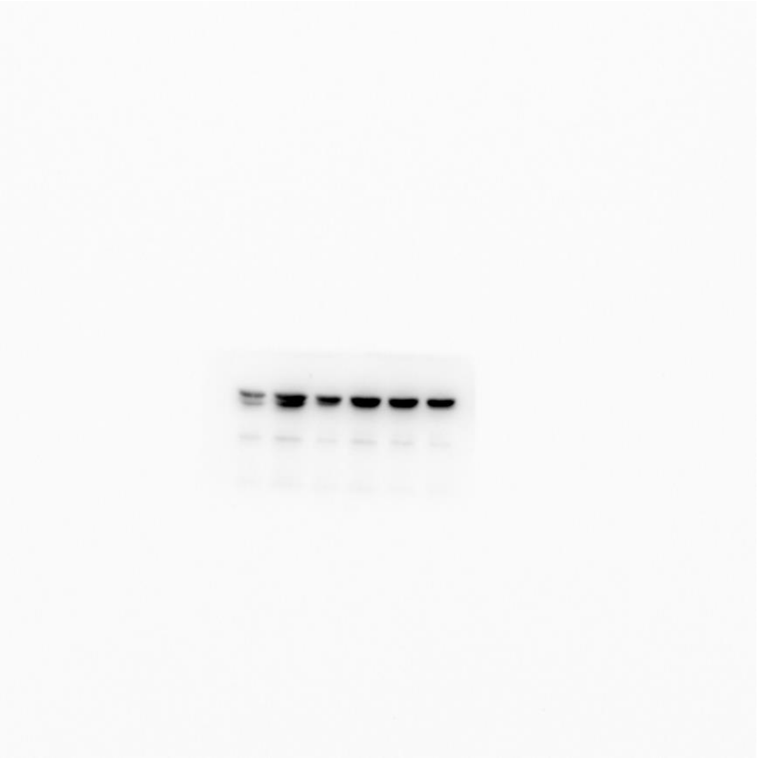

BAX

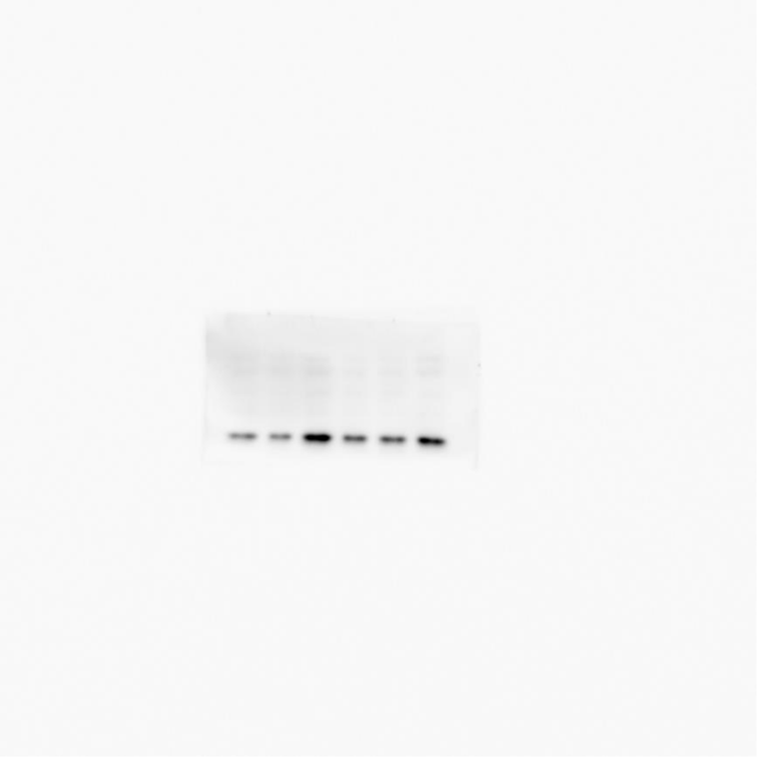

DDIT3

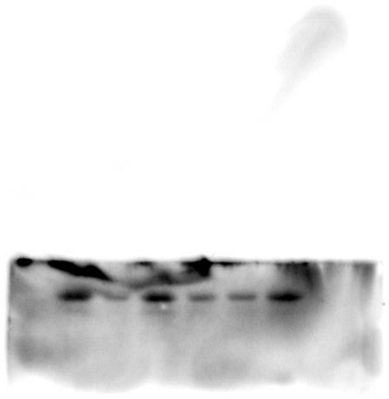

GAPDH

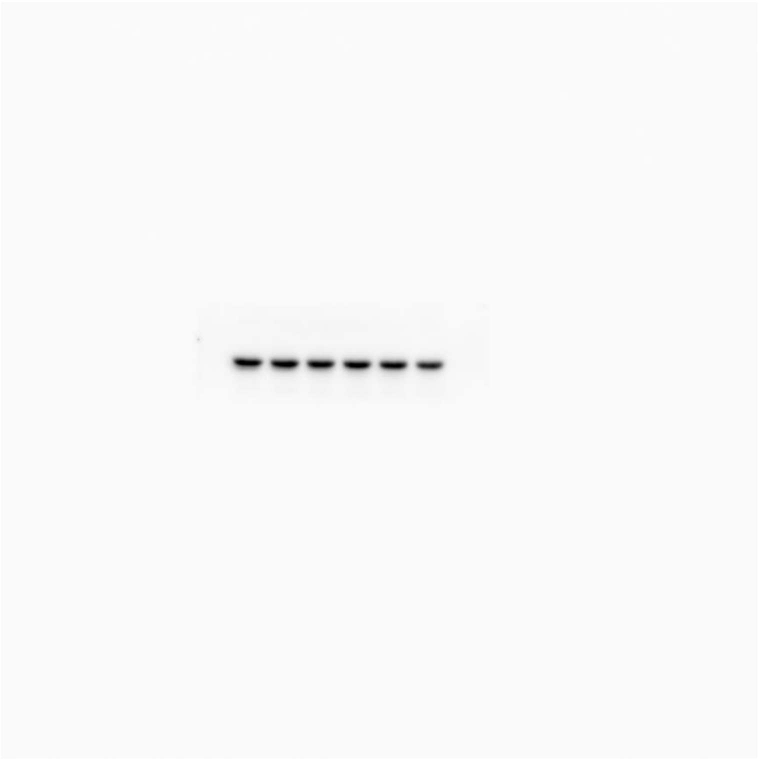

Figure 4E
BCL-2

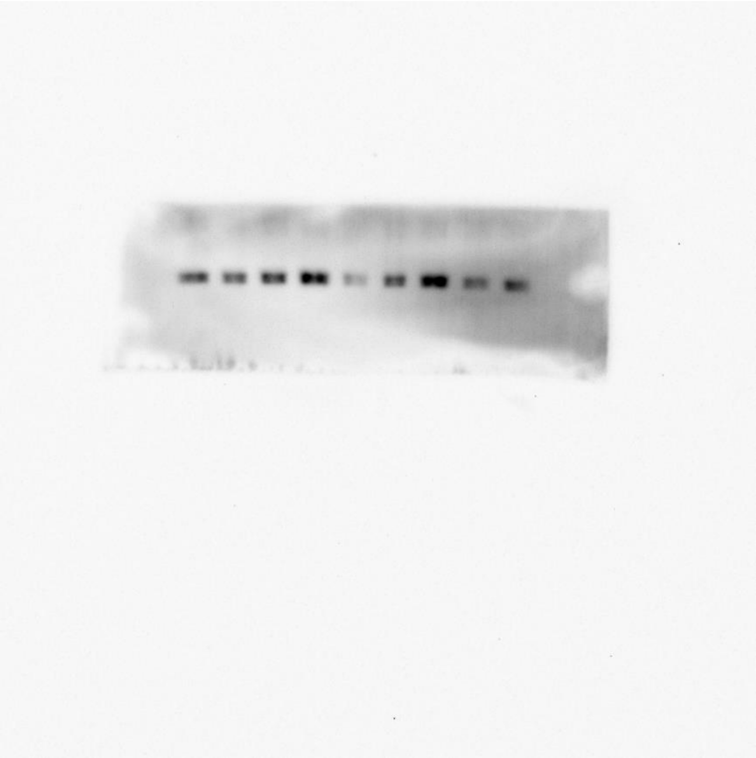

BAX

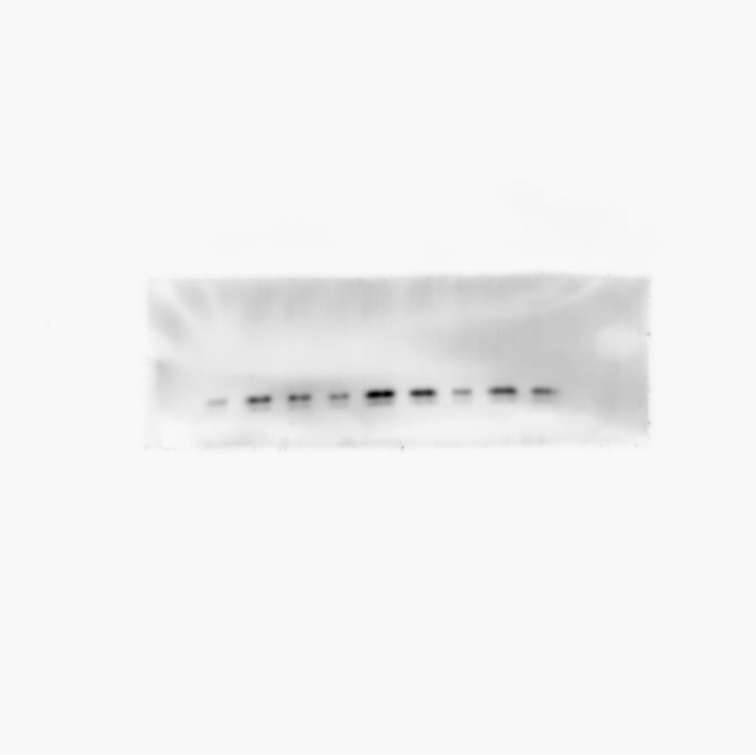

GAPDH

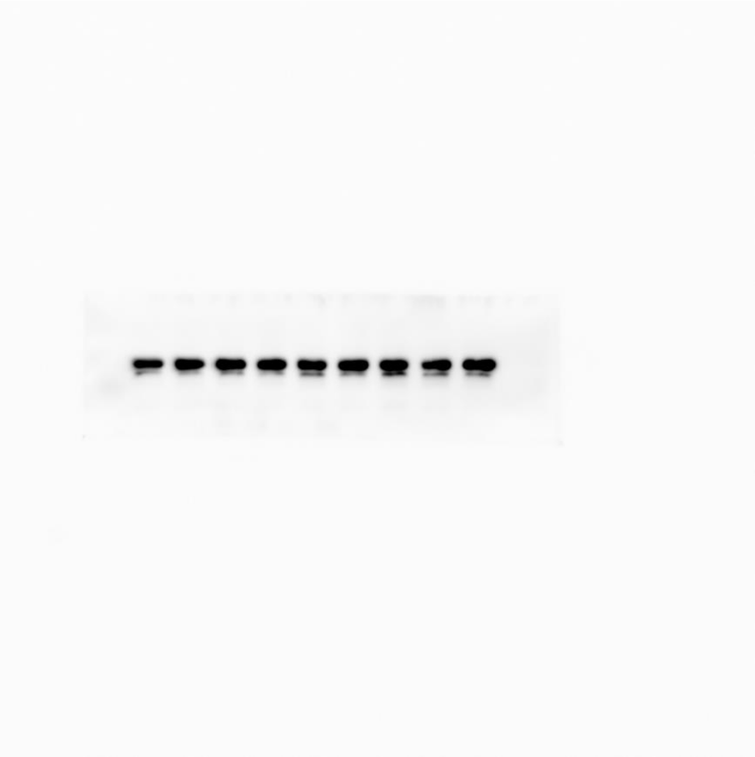

Figure 4F

Bcl-2

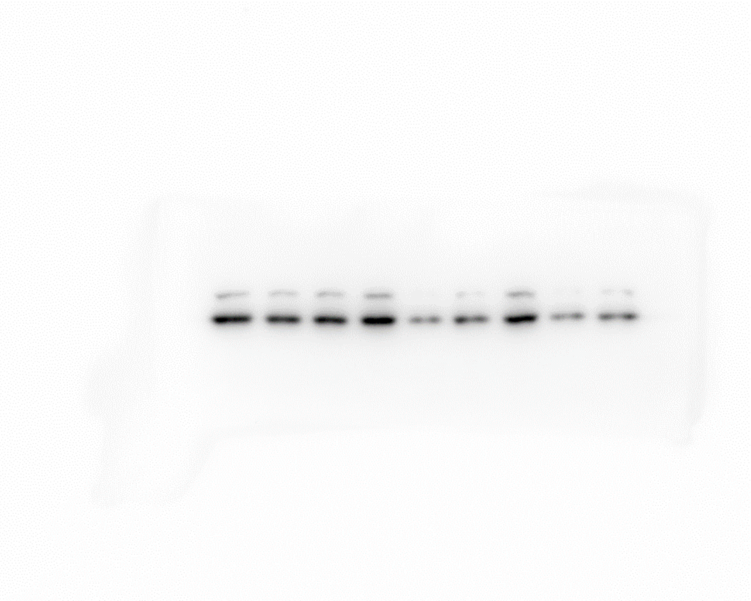

Bax

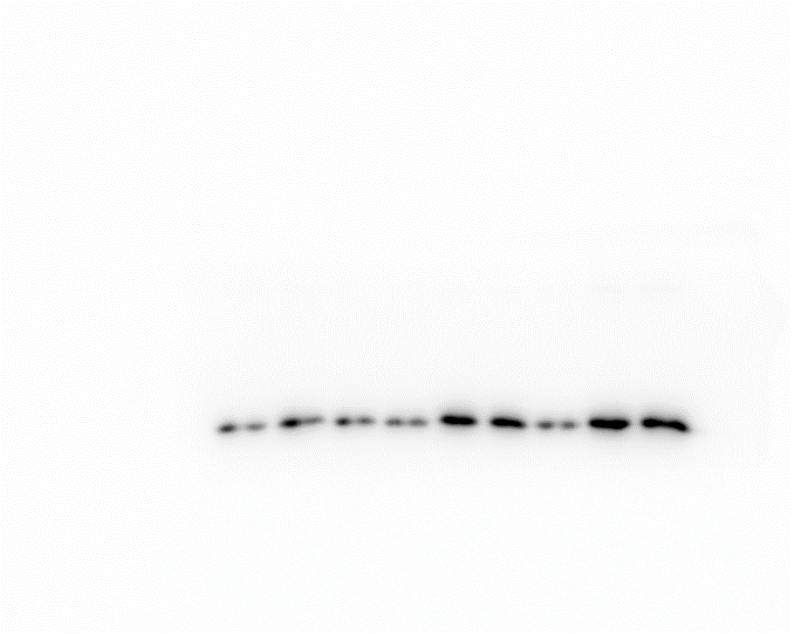

GAPDH

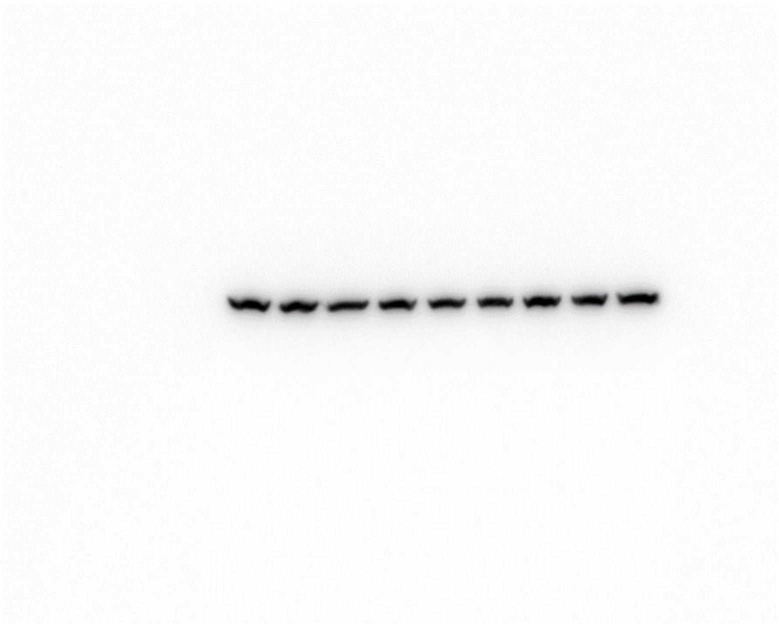

Figure 5B
BCL-2

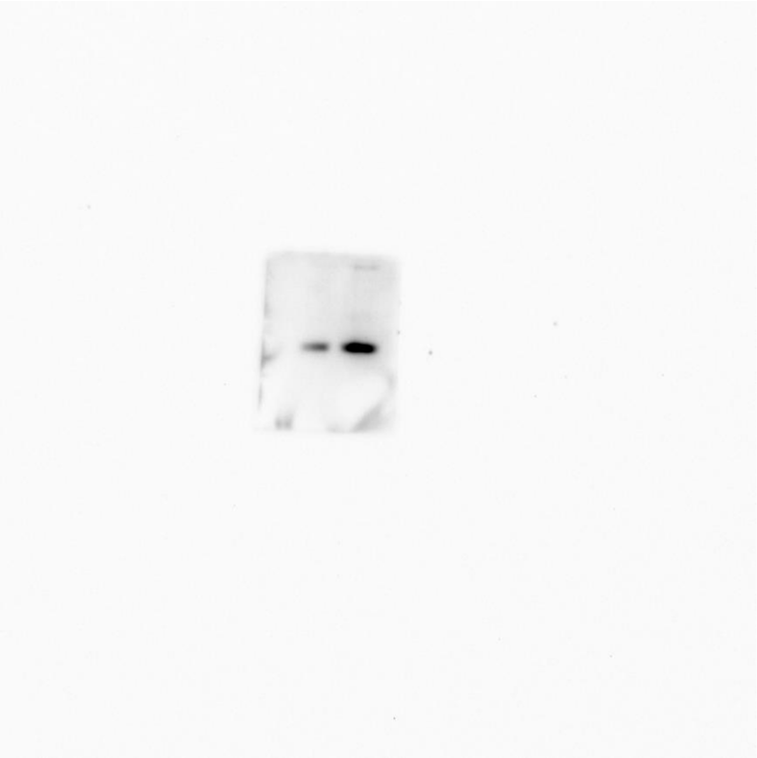

BAX

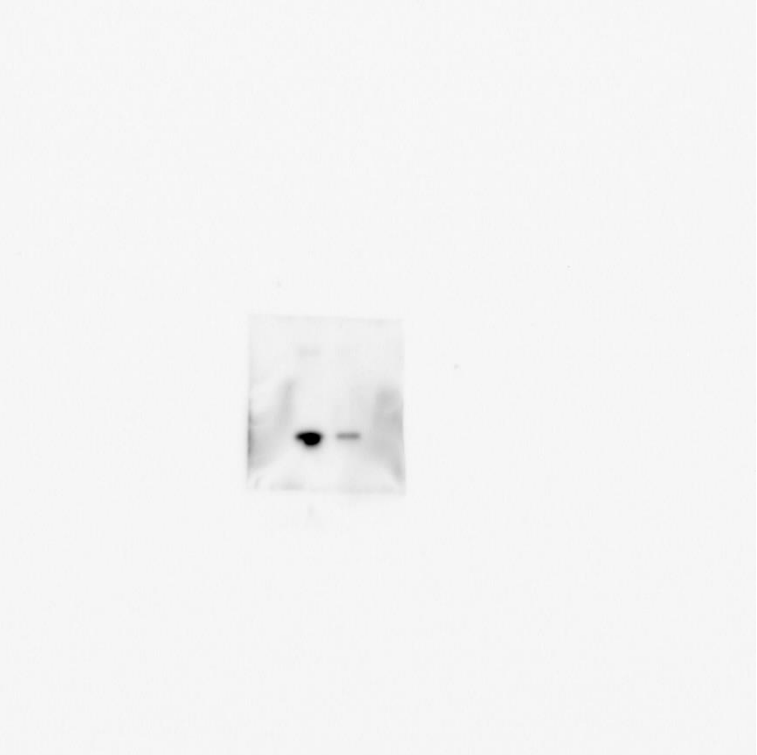

DDIT3

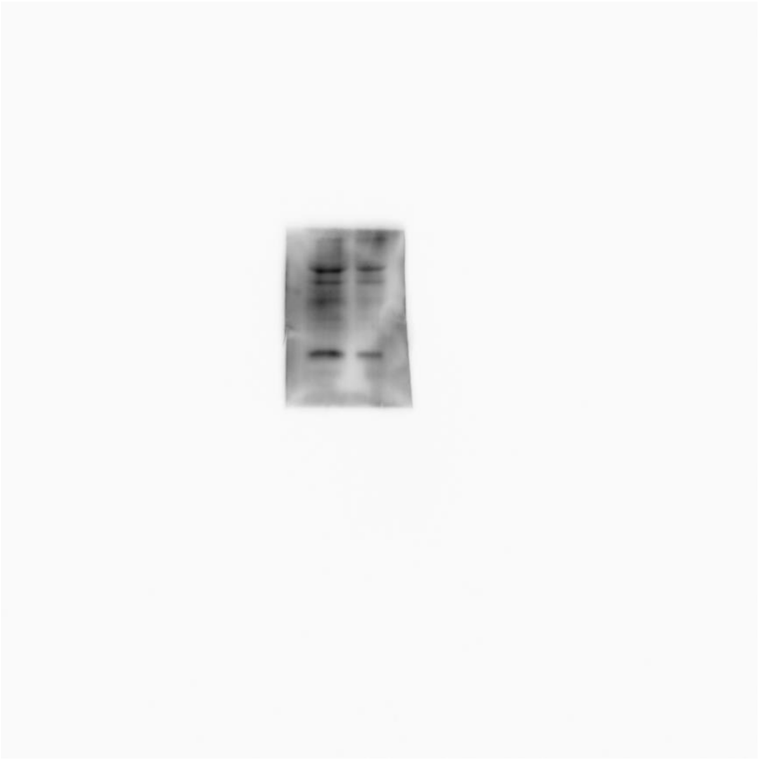

GAPDH

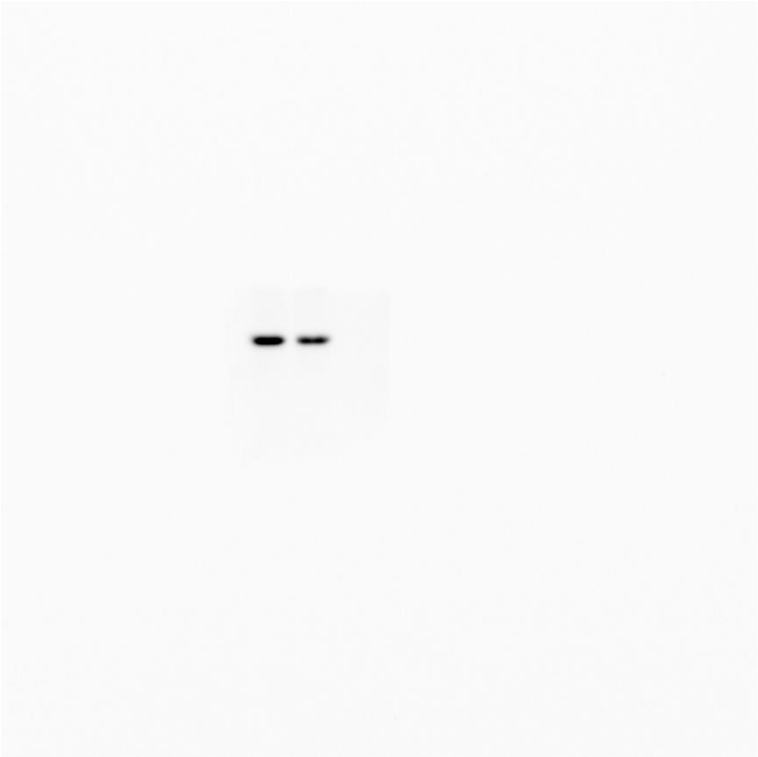

Supplementary Figure S3B

BCL-2

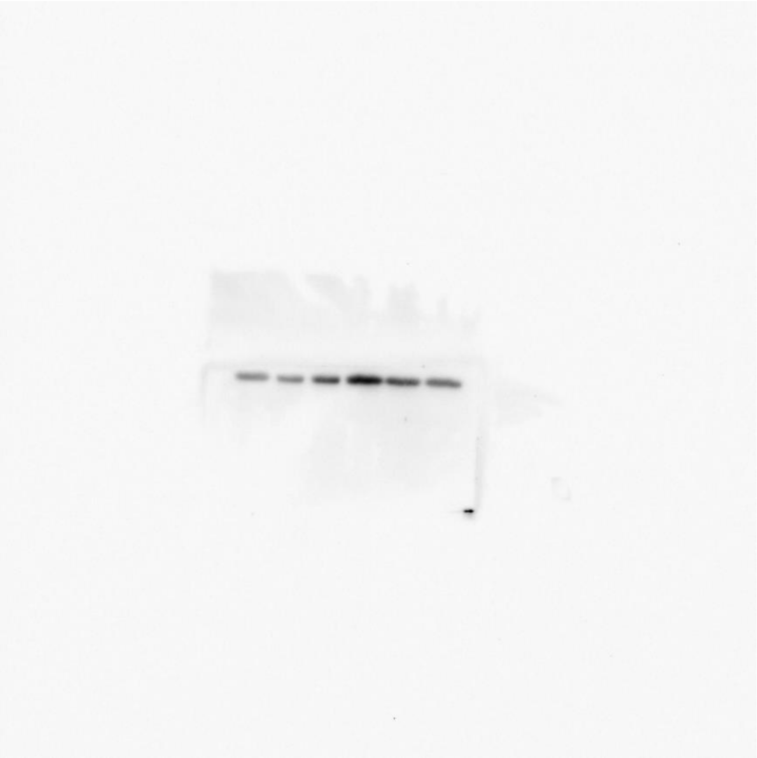

BAX

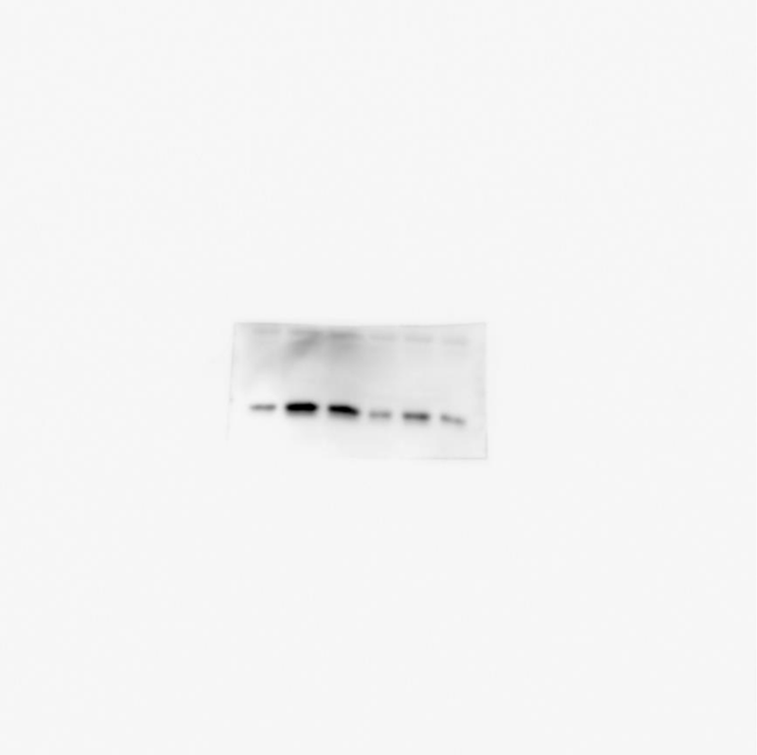

GAPDH

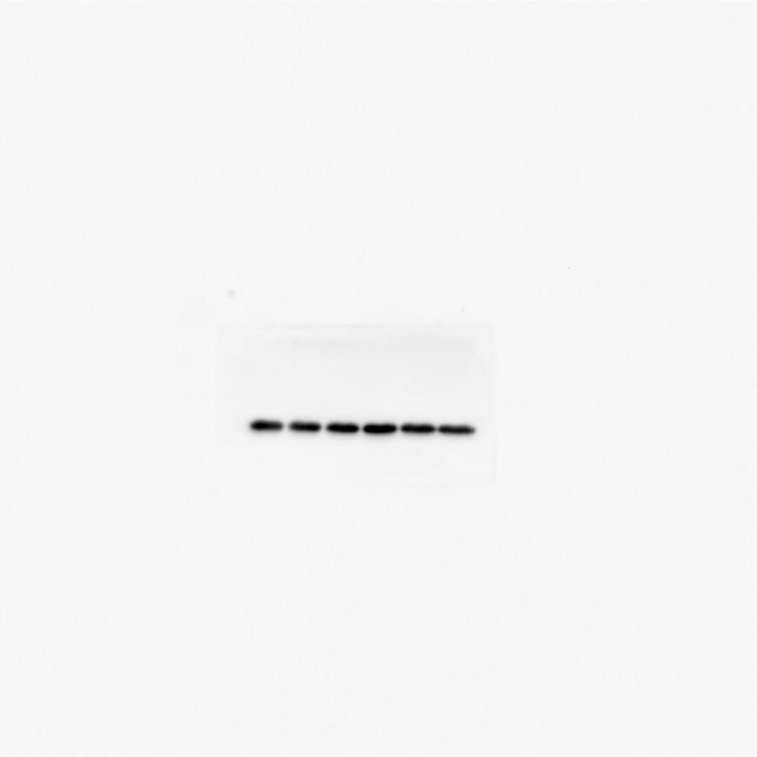

Supplementary Figure S4A

JAK2

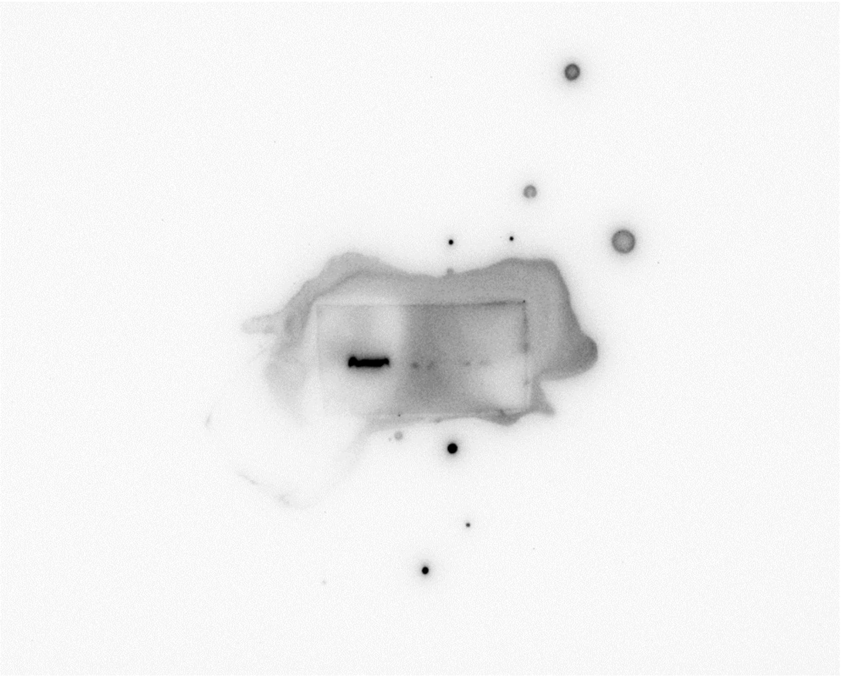

GAPDH

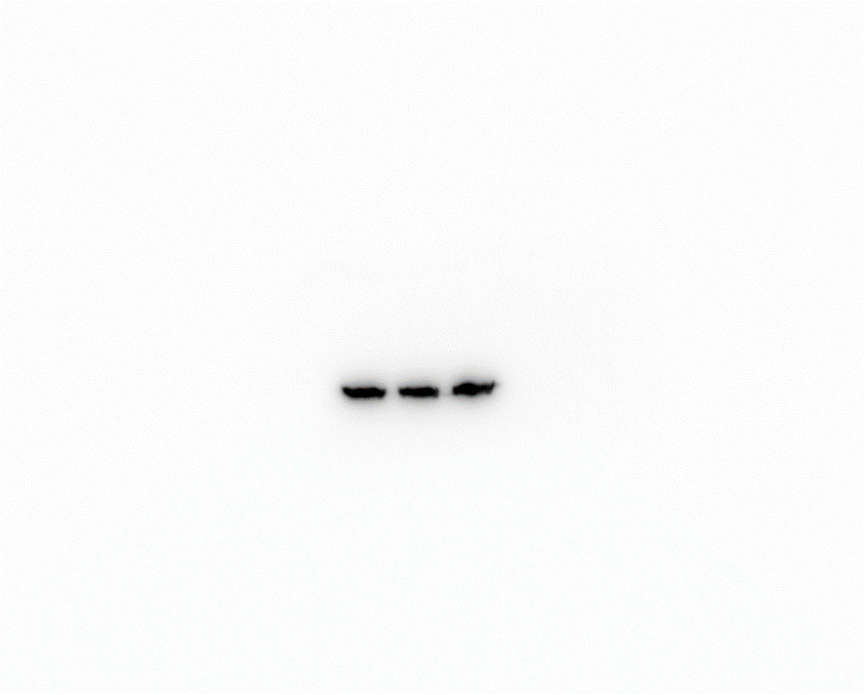

AKT

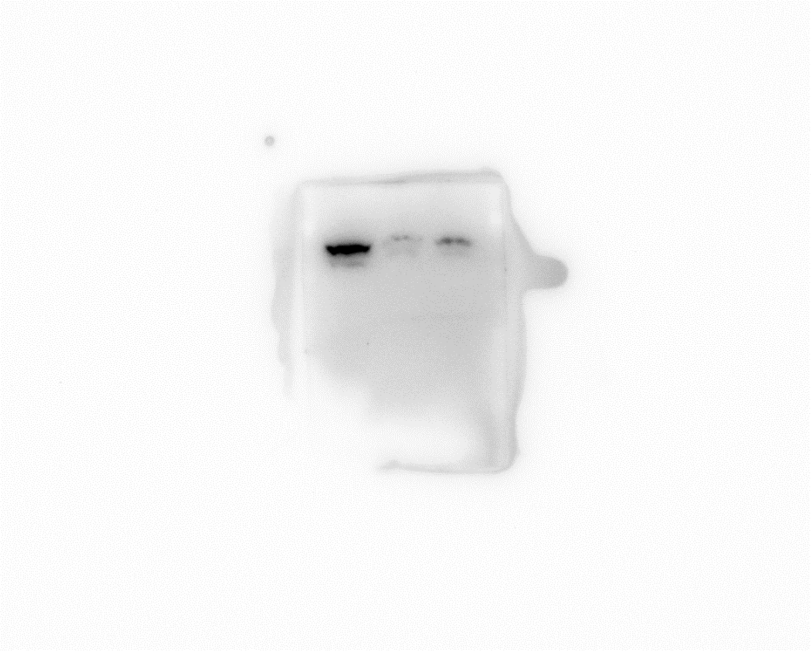

GAPDH

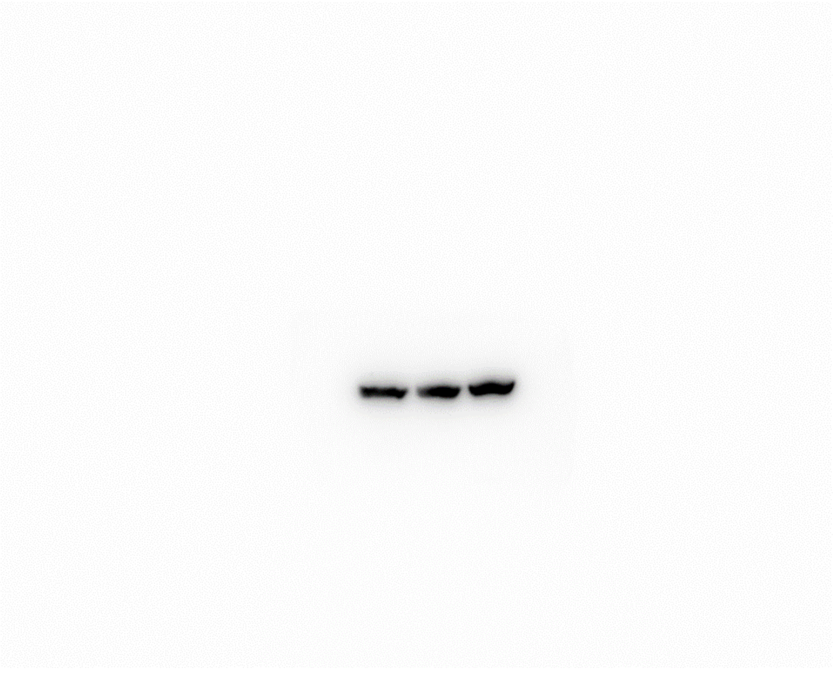

Supplement: Supplementary file 5 — Original western blots [file 41419_2022_5315_MOESM5_ESM.pdf]
