# Supplementary material for: c-Mpl-del, a c-Mpl alternative splicing isoform, promotes AMKL progression and chemoresistance
Source: Cell Death Dis. 2022 Oct 13;13(10):869. doi: 10.1038/s41419-022-05315-5 (PMC9561678; doi:10.1038/s41419-022-05315-5)
Supplement: Supplementary file 6 — Author Contribution Statement [file 41419_2022_5315_MOESM6_ESM.docx]

**Author Contribution Statement**

Fei Li designed and carried out experiments, analyzed data and prepared manuscript. Yuanyan Xiong and Mo Yang performed experiments, analyzed data and prepared the manuscript. Peiling Chen, Jingkai Zhang, Qiong Wang, Miao Xu, Yiming Wang, Zuyong He, Xin Zhao, Junyu Huang, Jingyao Li, Jinxin Ou, Ting Xu, Xueying Huang, and Yange Cao performed the research and analyzed data. Xiaoqiong Gu, Li Zhang, Rui Sun and Xunsha Sun collected and analyzed clinical data. Xiaohong Ruby Xu, Danielle Karakas, June Li and Heyu Ni designed experiments, analyzed data and wrote the manuscript. Qing Zhang supervised the research, carried out experiments, analyzed data and wrote the manuscript. All authors read and approved the final manuscript.
